# Supplementary material for: Street mothers’ well-being and motivation to leave street life in Bahir Dar city, Ethiopia: A phenomenological study
Source: PLoS One. 2022 Dec 15;17(12):e0278612. doi: 10.1371/journal.pone.0278612 (PMC9754257; doi:10.1371/journal.pone.0278612)
Supplement: S1 Appendix — (PDF) [file pone.0278612.s001.pdf]

S1 Appendix: In-depth interview guide (English and Amharic versions)

I. English version

I am ______________ working at BDU. Now, I would like to spend about 45-60 minutes with you to hear more about your experience while living on street with children. The purpose of this interview is to capture street mothers' experience of living on street. I will be asking you a range of questions and request you to answer questions in your own words. You can ask me to clarify if you have any questions about what is wanted. Please keep in mind that you may choose to not answer any question and that all information you provide will be confidential. You can also stop the interview at any time. However, your genuine response is very essential to understand the full picture of street mothers' life situation or motherhood on street including their well-being, their perception about their life on street and the motivation and efforts they tried to end street life. This in turn important to design intervention and strategies to improve the life situation of street mothers in the future.

If you need further information needed:

Contact person and address: Dabere Nigatu Tel: +251 913453579

Are you volunteer to participate?

1. Yes…continue
2. No…stop, thank you.

Do I have your permission to record our interview by audio-recorder?

1. Yes…start recording

2. No…stop interview

A) Background information

1. Code……………………………….
2. Interviewer ………………………...
3. Residence(previous)……………….
4. Age………………………………….
5. Educational status…………….
6. Marital status………………….
7. Street status……………………
8. Duration on the street………….
9. Number of children…………

B) Main Questions

1. How do you describe life on the street? (Probe: street status, economic status, income sources, life satisfaction)
2. How do you explain motherhood life on street? (Probe: self-care (feeding; hygiene, clothes, health care), child/ren care (looking after; feeding; hygiene, clothes, health care, education)
3. Please explain current physical wellbeing? (Probe: your physical wellbeing, your child's physical status)
4. Please describe your relationship with friends, families (partner), society? (Probe: your relationship, your child's relationship)
5. How do you describe your current mental/psychological/emotional well-being? (Probe: your mental, psychological or emotional wellbeing, your child's mental or psychological wellbeing)

6. How would you describe your current spiritual well-being? (Probe: your spiritual wellbeing, spiritual practice, your child’s spiritual practice)
7. What are the challenges you encounter while living or working on the street? (Probe; abuse (sexual, physical, psychological), stigma and discrimination (please give me examples), illness)
8. What do you think to leave street life? (Probe: desire, motivation to end street life)
9. What efforts you have tried to end street life? (Probe: for you, your child/ren, give example)
10. Describe the supports that you need to end street life? (Probe: from gov’t, community, NGO, private organization, religious organization)
11. Do you have additional information about this issue? If so please tell me.

Thank you for giving me your precious time!

II. Amharic version

የቃለ መጠይቅ መመሪያ ጥያቄዎች

እኔ ________________ እባላለሁ፤ ባህር ዳር ዩኒቨርሲቲ ውስጥ እየሰራሁ ነው። አሁን፣ ከልጆች ጋር በጎዳና ላይ ስትኖሩ ስላለው ህወት ልምድ የበለጠ ለመስማት ከ45-60 ደቂቃ ያህል ከእርስዎ ጋር ማሳለፍ እፈልጋለሁ። የዚህ ቃለ መጠይቅ አላማ የጎዳና ላይ እናቶችን የጎዳና ህይወት ልምድ ለማወቅና ለመተንተን ነው። የተለያዩ ጥያቄዎችን እጠይቅዎታለሁ እና ጥያቄዎችን በራስዎ ቃላት እንዲመልሱ እጠይቃለሁ ። ስለሚጠየቁት ነገር ማንኛውም አይነት ጥያቄ ካሎት እንዲብራራልኝ ብለው መጠየቅ ይችላሉ። እባክዎ ማንኛውንም ጥያቄ ላለመመለስ መምረጥ እንደሚችሉ እና ያቀረቡት ወይም የሚሰጡን መረጃ ሁሉ ሚስጥራዊ እንደሚሆን ያስታውሱ። እንዲሁም ቃለ መጠይቁን በማንኛውም ጊዜ ማቆም ይችላሉ። ነገር ግን፣ የጎዳና ላይ እናቶች ህይወት ሁኔታ ወይም እናትነት ማለትም በጎዳና ላይ ያሉ እናቶች ጤንነታቸውን፣ በጎዳና ላይ ስላላቸው ህይወት ያላቸውን ግንዛቤ እና የጎዳና ላይ ህይወትን ለማብቃት/ለማቆም ያደረጉትን ተነሳሽነት እና ጥረትን ጨምሮ የአንተ እውነተኛ ምላሽ በጣም አስፈላጊ ነው። ይህ ደግሞ ወደፊት የጎዳና እናቶችን የኑሮ ሁኔታ ለማሻሻል ስራዎችን ለመስራት ብሎም ስልቶችን ለመንደፍ አስፈላጊ ነው።

ተጨማሪ መረጃ ከፈለጉ፡- የዳበረ ንጋቱ አድራሻ፡- +251 913453579

ለመሳተፍ ፈቃደኛ ነዎት?

1. አዎ… ቀጥል።

2. አይ… አቁም፣ አመሰግናለሁ።

ቃለ ምልልሳችንን በድምጽ መቅጃ ለመቅዳት ፍቃድህ አለኝ?

1. አዎ… መቅዳት ጀምር

ሀ) ቅድመ መረጃ

1. ኮድ ……………………………….

2. ቃለ መጠይቅ አድራጊ ………………

3. የመኖሪያ ቦታ (የቀደሞ) …………….

4. ዕድሜ ………………………………

5. የትምህርት ደረጃ ………………….

6. የጋብቻ ሁኔታ ………………………

7. የማደሪያ ቦታ ………………………

8. በጎዳና ላይ የቆዩበት ጊዜ ………….

9. የልጅ ብዛት ________________________________

ለ) ዋና ጥያቄ

1. የጎዳና ሕይወትዎን እንዴት ይገልጹታል? (**ፍንጭ** ፣ የጎዳና ኑሮ ሁኔታ ፣ ኢኮኖሚያዊ ፣ የሕይወት እርካታ ምሳሌዎችን መስጠት ይችላሉ)
2. በጎዳና ላይ የእናትነት ሕይወትዎን እንዴት ይገልጹታል? (**ፍንጭ** -ራስን መንከባከብ (መመገብ ፣ ንፅህና ፣ ልብስ ፣ የጤና እንክብካቤ) ፣ የልጆች / ሕፃናት እንክብካቤ (መንከባከብ ፣ መመገብ ፣ ንፅህና ፣ አልባሳት ፣ የጤና እንክብካቤ ፣ ትምህርት)
3. አካላዊ ደህንነትዎን እንዴት ይገልጹታል? (**ፍንጭ፡** የእርስዎን አካላዊ ደህንነት ፣ የልጅዎን/ልጆችዎን አካላዊ ደህንነት)
4. ከጓደኛ ፣ ከቤተሰብ (ኢጋር) ፣ ከህብረተሰብ ጋር ያለዎትን ግንኙነት እንዴት ይገልጹታል? (**ፍንጭ፡** የእርስዎን ግንኙነት ፣ የልጅዎ / ልጆችዎ ግንኙነት)
5. የአእምሮ/የስነልቦና ደህንነትዎ እንዴት ነው? (**ፍንጭ፡** እርስዎ የአእምሮ/የስነልቦና ደህንነት ፣ ልጅዎ/ልጆችዎ የአእምሮ/የስነልቦና ደህንነት)
6. መንፈሳዊ ደህንነትዎን እንዴት ይገልጹታል? (**ፍንጭ፡** የእርስዎን መንፈሳዊ ደህንነት፣ መንፈሳዊ ድርጊቶች፣ የልጅዎ መንፈሳዊ ድርጊቶች)
7. በጎዳና ላይ ሲኖሩ ወይም ሲሰሩ ያጋጥምዎት ችግሮች ምንድን ናቸው? (**ፍንጭ** ፣ ጥቃት (ወሲባዊ ፣ አካላዊ ፣ ሥነ-ልቦናዊ) ፣ መገለል እና አድልዎ (እባክዎን ምሳሌዎችን ይስጡኝ) ፣ መቋቋሚያ ዘዴ)

8. የጎዳና ህይወትን ለመተው ምን ያስባሉ? (**ፍንጭ፡** ለማቆም ፍላጎት ፣ ተነሳሽነት)

9. የጎዳና ላይ ሕይወትን ለማቆም ምን ጥረት አድርገዋል? (**ፍንጭ፡** ለእርስዎ ፣ ለልጅዎ/ልጆችዎ፥ ምሳሌ ይስጡ)

10. የጎዳና ህይወትን ለማቆም የሚያስፈልጉዎትን ድጋፎች ይግለጹ? (**ፍንጭ፡** ከመንግስት ፣ ማህበረሰብ ፣ መንግስታዊ ያልሆነ ድርጅት ፣ የግል ድርጅት ፣ የሃይማኖት ድርጅት)

11. ስበጎዳና ላይ እናትነት ተጨማሪ መረጃ አለዎት? ከሆነ እባክዎን ንገሩኝ ፡፡

ውድ ጊዜዎትን ስለሰጡኝ አመሰግናለሁ!
