# Supplementary material for: Street mothers’ well-being and motivation to leave street life in Bahir Dar city, Ethiopia: A phenomenological study
Source: PLoS One. 2022 Dec 15;17(12):e0278612. doi: 10.1371/journal.pone.0278612 (PMC9754257; doi:10.1371/journal.pone.0278612)
Supplement: S2 Appendix — (PDF) [file pone.0278612.s002.pdf]

S2 Appendix: Thematic framework showing themes, subthemes and codes used during analysis

Themes	Subthemes	Codes
Well-being	Physical well-being	Illness<br>Swelling<br>Urine color change<br>Pusy urine<br>Illness duration<br>Emaciated<br>Leg stiffness<br>Abdominal distension<br>Mobility difficulty<br>Feeling numbness<br>Troubling pain<br>Unable to move<br>Eye redness<br>Unable to steep<br>Mental dullness<br>Brother sickness<br>Art user<br>Child disability<br>No health problem<br>Child sickness
	Social well-being	Family loss<br>Family attachment<br>Loneliness<br>Colleague disturbance<br>Colleague theft<br>Colleague conflict<br>Conflict resolution<br>Family status<br>Safeguard<br>Community attachment<br>Child relation<br>Family protection<br>Children's emotional support<br>Will of neighbors<br>Husband loss<br>Friend attachment<br>Husband relation
	Economic well-being	Poorness<br>Material support<br>Financial support<br>Food support<br>Clothes support<br>Income source<br>Low income for livelihood

		Market Inflation<br>Supporting childcare<br>Hygiene related economy
	Mental well-being	Worry<br>Stress<br>Suicidal ideation<br>Worry children's mental health<br>Children's worry<br>Forgetfulness<br>Anxiety<br>Depress
	Emotional well-being	Fear of accident<br>Hopelessness<br>Feels sad<br>Tearing<br>Children's worry<br>Fear of getting disease<br>Fear of children loss<br>Anger<br>Fear for life<br>Feeling
	Spiritual well-being	Traditional treatment<br>Hope in God<br>Thanking God<br>Attending holy water<br>Praying<br>Promise to God<br>Joining monasteries<br>Blood-flesh<br>Religious support<br>God will<br>Christian life
Perception	Perception	Fear of accident<br>Child protection<br>Societal perception<br>Child misbehaves<br>Perception<br>Need adult protection<br>Child suffering<br>Perceived children's life<br>Child independence<br>Inquire why on street<br>Family protection<br>Fear of getting disease<br>Perceived risks<br>Poor not heard

		Fear of children loss<br>Fear for life<br>Patience<br>Perceived severity
Motivation	Motivation to leave	Desire for education<br>Education for reinforcement<br>Desire to leave<br>Giving child to relative<br>Joining monasteries<br>Desire to make money<br>Desire to work<br>Desire to own house<br>Suggest change living place<br>Support needed to leave<br>Investing on children<br>Desire for self-contained life
	Efforts to leave	Child education<br>Effort to end street<br>Giving child to relative<br>Child work
