# Supplementary material for: Street mothers’ well-being and motivation to leave street life in Bahir Dar city, Ethiopia: A phenomenological study
Source: PLoS One. 2022 Dec 15;17(12):e0278612. doi: 10.1371/journal.pone.0278612 (PMC9754257; doi:10.1371/journal.pone.0278612)
Supplement: S1 Dataset — (PDF) [file pone.0278612.s003.pdf]

Dataset

Synthesis 1: Physical well-being

LineNo	Text	Codes	Synthesis 1
	Document: IDI-Translation_Compiled		
23	sleeping area is better. I lost my health before my husband	illness	Physical well-being
24	died. My side swells and my urine turn into pus. Then it	pusy urine, swelling, urine color change	Physical well-being
25	[swelling] came to my neck. I get sick like that for five	swelling	Physical well-being
26	years ago. As it started, I went to Gishen DebreKerbe	Illness duration, swelling, traditional treatment	Physical well-being, Spritul well
39	drinking ten liters Tsebel a day and I'm too emaciated. God	Emaciated, hope in God, traditional treatment	Physical well-being, Spritul well
41	returned, I suspected they crushed them [children]. It's a	fear of accident, leg stiffness	Emotional well-being, Perceptio
42	year it tied my leg. When it tied my leg, I would have	abdominal distension, leg stiffness	Physical well-being
43	blown stomach. It looks like a sack that encloses with a	abdominal distension	Physical well-being
44	drawstring.	abdominal distension	Physical well-being
46	Respondent: It swells up (she is pointing to the left leg).	swelling	Physical well-being
47	This shrink, swells in this direction (she is pointing to	swelling	Physical well-being
48	the right leg) and my urine, uterus, and urine were changed.	urine color change	Physical well-being
49	When it swells, the swelling extends up. I don't have a	family loss, swelling	Physical well-being, Push facto
70	(she is pointing the waist), I look like normal. But it is	mobility difficulty, societal perception	Perception, Physical well-being
71	difficult for me to move and walk easily. I believe that	desire for education, mobility difficulty	Motivation to leave, Physical w
108	Respondent: This left leg (pointing to the left foot)	Feeling Numbness	Physical well-being
109	starting from head in the left side, it is not seeming for	Feeling Numbness	Physical well-being
110	mine. The illness is changeable. Now, it [illness] tied leg,	Feeling Numbness	Physical well-being
111	"Yekodekudegnal", let my feet tremble. It kicks me in the	troubling pain	Physical well-being
112	leg. Above that, it cached, this, my stomach (she is	troubling pain	Physical well-being
113	pointing to stomach), it breaks my waist. And when it	troubling pain, unable to move	Physical well-being
114	breaks my waist, I can't swing like this. My stomach swells	unable to move	Physical well-being
115	up. Then, my urine changes.	unable to move, urine color change	Physical well-being
117	Respondent: My urine turns into pus. When it swells, it	pusy urine	Physical well-being
118	turns into pus. My urine stinks; It turns into pus. My	pusy urine	Physical well-being
119	urine smells bad. And like that it is changing, I'm worried	pusy urine, Worry	Mental well-being, Physical well
121	When I get stressed, my eyes change. It looks like red. I'm	eye redness, stress	Mental well-being, Physical well
122	stressed. I don't sleep on my left side. When I was about	sleeping disturbance, stress, troubling pain	Mental well-being, Physical well
123	to fall asleep (she is leaning to her side) in the left	sleeping disturbance, troubling pain	Physical well-being
126	Respondent: The one that it clings to my feet is the one	swelling	Physical well-being
127	that swells and swells and then move to swells and swells	swelling	Physical well-being
128	in the other area. This whole leg cannot be rushed. This is	swelling, unable to steepon	Physical well-being
129	not going to work. when I step on it, my ligament (she is	unable to steepon	Physical well-being

LineNo	Text	Codes	Synthesis 1
130	pointing to the left foot) does not allow me to go, just	unable to move	Physical well-being
131	like tied things. And it irritates me, “yebelagnal”. It	unable to move	Physical well-being
132	bullies me by using knife, “Karawon yezo new yemisekeskegn”.	troubling pain	Physical well-being
134	Respondent: It irritates, “yesenezregnal”, me. As it rises,	troubling pain	Physical well-being
135	hurts my ligaments and ligaments. Due to pain, I did not	troubling pain	Physical well-being
136	step on the ground. This great toe (she is pointing to the	troubling pain	Physical well-being
137	left great toe) touches. Half of my great toe treads on the	unable to steepon	Physical well-being
138	ground. But I will not be trampled underfoot.	unable to steepon	Physical well-being
163	Respondent: My mind is blured. I sat in St. George in a	mental dullness	Physical well-being
367	Respondent: I am fine. There is no pain. And children are	no health problem	Physical well-being
368	healthy.	no health problem	Physical well-being
516	Respondent: We are still safe. We have never been sick. We	no health problem	Physical well-being
517	don't have any problems.	no health problem	Physical well-being
687	Respondent: previously she was sick, I bought syrup from	illness, modern treatment	Basic need related, Physical w
690	Respondent: No, I have not encountered any sickness.	no health problem	Physical well-being
695	Respondent: if I am not sick, I assume I'm fine. When my	no health problem	Physical well-being
696	child is laughing like this, I assume she is fine too. My	no health problem	Physical well-being
697	child and I myself are safe. But, sometimes I would say	no health problem	Physical well-being
698	that my daughter is sick if she is not playing.	no health problem	Physical well-being
904	I sleep on the street since this my arm has broken.	illness, perception	Perception, Physical well-being
906	Respondent: Do you understand what I mean? Now I am here	illness, push factor	Physical well-being, Push facto
911	Respondent: I fall. I was broken when I fall on the street.	illness	Physical well-being
912	It is a year ago.	Illness duration	Physical well-being
940	Respondent: my physical health, Ere (??)… I'm fine. Up to	no health problem	Physical well-being
976	Respondent: Ere (??)…, I'm fine. Just I am here [on the	no health problem, perception	Perception, Physical well-being
1077	before this time. Thanks to God, I am fine till now.	no health problem, Thanking God	Physical well-being, Spritul well
1126	Respondent: He is not considered as he is living. First of	Family attachment, illness	Physical well-being, Social well
1127	all, he has a falling disease. When he feels better, he	Family attachment, illness	Physical well-being, Social well
1128	comes to the home but when he is sick, he may not come to	Family attachment, illness	Physical well-being, Social well
1129	home and spend the night outside. Since he is suffering	Family attachment, illness	Physical well-being, Social well
1130	from a mental illness, he is not considered as he is coming	Family attachment, illness	Physical well-being, Social well

LineNo	Text	Codes	Synthesis 1
1131	to home.	Family attachment, illness	Physical well-being, Social well
1133	Respondent: it drops him. He usually swallows a pill. When	illness	Physical well-being
1134	he falls, the foam is seen on his nose and mouth. Even if	illness	Physical well-being
1135	he comes home, he will not help me 100%. What he helped me?	illness	Physical well-being
1136	If you think as if I lie, anybody can be asked from	illness	Physical well-being
1137	neighbor and can be confirmed. My helpers are the God and	illness	Physical well-being
1138	only these children.	illness	Physical well-being
1174	Respondent: Ere…, I'm fine, thanks to God. There is some	no health problem, Thanking God	Physical well-being, Spritul well
1175	problem on my eye; otherwise, I'm fine, thanks to God. One	illness, Thanking God	Physical well-being, Spritul well
1176	of my eyes is tearing. I have never been go to a hospital,	illness, modern treatment	Basic need related, Physical w
1205	Respondent: Yes, previously she was very sick with Yesamba	illness	Physical well-being
1206	mich (???? ??).	illness	Physical well-being
1208	Respondent: she was severely coughing. She was sick and	illness	Physical well-being
1209	treated for anemia (?? ??? ???? ??????????).	illness	Physical well-being
1458	Answer: The child is sick. His body is fine. But I do not	child sickness	Basic need related, Physical w
1459	know what hurts him, he is crying 24 hours.	child sickness	Basic need related, Physical w
1461	Answer: yes	child sickness	Basic need related, Physical w
1464	When I ran out of money, I never took it. Let him grow up	child sickness, modern treatment	Basic need related, Physical w
1465	if God allow the child for me; if not, it doesn't matter to	child sickness, modern treatment	Basic need related, Physical w
1466	me. His body is fine. But sometimes he(her child) has	child sickness, modern treatment	Basic need related, Physical w
1467	diarrhea.	child sickness, modern treatment	Basic need related, Physical w
1469	Answer: There is nothing else. He cries. I don't know his	child sickness	Basic need related, Physical w
1470	illness.	child sickness	Basic need related, Physical w
1511	Answer: My son is sick. If I am lucky, he will be growing	child sickness	Basic need related, Physical w
1512	up for me, if no, I can’t do anything to cure from the	child sickness, hope in God, hopelessness	Basic need related, Emotional
1513	illness.	child sickness, hope in God, hopelessness	Basic need related, Emotional
1597	My child was sick. My relatives helped by the government.	child sickness	Basic need related, Physical w
1756	Besides, I have a sick brother. He is as sick as I am. No	brother sickness, Health care	Basic need related, Physical w
1757	one takes care of him himself; he is unemployed and moves	brother sickness, lack of support	Physical well-being
1758	here and there. I let him live with me instead of he being	brother sickness, lack of support	Physical well-being
1769	Respondent: My wellbeing, I am user; I have nothing else. I	ART user	Physical well-being
1770	use a drug.	ART user	Physical well-being

LineNo	Text	Codes	Synthesis 1
1772	Respondent: I use a drug for the virus. No other thing	ART user	Physical well-being
1773	other than that [the virus] at this time (with a speech	ART user	Physical well-being
1774	signaling hopelessness). So far, I survived here because of	ART user, God will	Physical well-being, Spritul well
1864	living with me because he found hard to leave me here. But	brother sickness, Family attachment	Physical well-being, Social well
1865	he is weak, he is sick, he has nothing, if I got, he will	brother sickness	Physical well-being
1866	eat. We used to eat this [monthly salary] just for five	brother sickness, Income source	Economical well-being, Physica
2075	Respondent: It has been long since he has been informed of	brother sickness	Physical well-being
2076	having liver disease "Yewefua".	brother sickness	Physical well-being
2078	Respondent: Yes, they said and he is sick. On top of that,	brother sickness, support	Physical well-being
2160	compared with assets. These children; first this child's	child disability, God will, Value life than assets	Physical well-being, Spritul well
2161	[little child] disability that God make him lost his body.	child disability, God will	Physical well-being, Spritul well
2180	grows for me. But now, he unable to maintain upright	child disability, desire for education	Motivation to leave, Physical w
2181	position and walk/go. Thank God for another problem.	child disability, desire for education, Thanking God	Motivation to leave, Physical w
2184	Respondent: I'm fine. I have nothing and never, it is this	child disability, no health problem	Physical well-being
2185	child who bothered me otherwise I can work and eat.	child disability	Physical well-being
2188	Respondent: Only his eye, his eye.	child disability	Physical well-being
2190	Respondent: Only his eye.	child disability	Physical well-being
2192	Respondent: His eyes do not see; Can't you see?	child disability	Physical well-being
2194	Respondent: Look him, there is no problem (she uncovered	child disability	Physical well-being
2195	the baby while he is on her back. Both eyes cannot see; the	child disability	Physical well-being
2196	eye balls are not present; they are blind)	child disability	Physical well-being
2198	Respondent: Off course; both eyes are from birth.	child disability	Physical well-being
2200	Respondent: Three	child disability	Physical well-being
2202	Respondent: Yes, 3 years is over now.	child disability	Physical well-being
2204	Respondent: He cannot. He unable stand. He is just a blind	child disability	Physical well-being
2205	that he unable stand, that is all.	child disability	Physical well-being
2223	Respondent: She [the older daughter] is fine. Nothing.	no health problem	Physical well-being
2236	Respondent: Nothing, I am fine	no health problem	Physical well-being

LineNo	Text	Codes	Synthesis 1
2238	Respondent: Children; except this one [little child] that	no health problem	Physical well-being
2239	one [older child] is safe.	no health problem	Physical well-being

Synthesis 1: Social well-being

LineNo	Text	Codes	Synthesis 1
	Document: IDI-Translation_Compiled		
15	Respondent: I don't have a mother or a father. It was my	Family attachment, family loss	Push factors, Social well-being
16	grandmother who raised me and let me married. And when my	Family attachment, family loss	Push factors, Social well-being
17	husband died, I took a home with my money and spent it on	Asset loss, family loss	Push factors, Social well-being
18	medicines and tsebel. When he died and his father was on	family loss, Health care	Basic need related, Push factors, Soc
49	When it swells, the swelling extends up. I don't have a	family loss, swelling	Physical well-being, Push factors, So
50	mother or a father. What can I do to help children? I gave	family loss, hopelessness	Emotional well-being, Push factors, S
52	hits his eye. who do I have? When a child goes crazy	child beat, loneliness	Physical abuse, Social well-being
141	Respondent: The people here are “tsebeltegna”. Some are	Colleague disturbance	Social well-being
142	insane. There are those who lose their minds and are	Colleague disturbance	Social well-being
143	baptized. It means they have no control over anything.	Colleague disturbance	Social well-being
144	There are also day laborers who work and eat here. Within	Colleague disturbance	Social well-being
145	the tsebel house, there are individuals who involved in	Colleague disturbance	Social well-being
146	disturbance, “meneshere”.	Colleague disturbance	Social well-being
148	Respondent: It means to fight. They simply fight with the	Colleague disturbance	Social well-being
149	“tsebeltegna”. They clash. Those who are mentally retarded	Colleague disturbance	Social well-being
150	are easily fed by “tsebeltegna”. For example, there was a	Colleague disturbance	Social well-being
151	woman who lost her mind. On the way, when she goes out	Colleague disturbance	Social well-being
152	someone returns her. Those who are angry with house rent	Colleague disturbance	Social well-being
153	live in the “Tsebel” house and do their daily work at day	Colleague disturbance	Social well-being
154	time. They spend the night in “tsebel” house. Some have	Colleague disturbance	Social well-being
155	children.	Colleague disturbance	Social well-being
174	Respondent: I always live with confusion. Because I have no	loneliness	Social well-being
175	one. Ehh, just I'm falling on the street at this age. Ehh,	loneliness, perception	Perception, Social well-being
197	hundred and forty birr. I lost that one hundred and forty	colleague teft	Social well-being
198	birr, where I put it in the “tsebel” house, they stole it	colleague teft	Social well-being
199	and I lost it. I cried and kept quiet. A woman from there	colleague teft, Food support	Basic need related, Economical well-
202	that. The other day I gave it to her and she hit me, saying	colleague conflict, Hit	Physical abuse, Social well-being
203	you called me a thief. She hit me hard. Then I told them	colleague conflict, Hit, Insult	Physical abuse, Psychological abuse,
205	handicap doesn't sit here. I brought the police and showed	conflict resolution, Insult	Psychological abuse, Social well-bein
206	her the woman. He told he Don't bother her again, and he	conflict resolution	Social well-being
207	also said to me what do you want me to do for you? I told	conflict resolution	Social well-being
208	him not to hit me, I just wanted to be reconciled. Then I	conflict resolution	Social well-being
209	reconciled her.	conflict resolution	Social well-being

LineNo	Text	Codes	Synthesis 1
378	Respondent: If I have, for example, we will share the night	Friend attachment	Social well-being
379	together. I live in a rented house and she lives of street	Friend attachment, sleeping place	Basic need related, Social well-being
380	(she is pointing to her friend). So, I tell her to come and	Friend attachment, sleeping place	Basic need related, Social well-being
381	spend the night together until the rain stopes. Together,	Friend attachment	Social well-being
382	mutually helping each other. If I haven’t, she feed my	Friend attachment	Social well-being
383	children. If she hasn’t, I feed her child. Sometimes I do	Friend attachment	Social well-being
384	not get food. Most of the time, I am hurting myself because	Friend attachment	Social well-being
520	Respondent: Some individuals in the street are nice. But we	Friend attachment	Social well-being
521	street individuals, if one has not had food, the other	Friend attachment	Social well-being
522	feeds him, and vice versa. For example, if the woman here	Friend attachment	Social well-being
523	has no money, I will give it to her. Or they [children] may	Friend attachment	Social well-being
524	eat by sharing. Or I buy something for the baby [her baby],	Friend attachment	Social well-being
525	it doesn't matter if we fast. And we are happy to share it,	Friend attachment	Social well-being
546	government. I also did not give him; I want to raise my	lack of family support, loneliness	Social well-being
547	child.	lack of family support, loneliness	Social well-being
551	the guards do not let us sleep. They get up at nine o'clock	Colleague disturbance	Social well-being
552	and wake us up. Then we slept in front of it at any place.	Colleague disturbance, sleeping place	Basic need related, Social well-being
553	We get up at twelve o'clock in the morning.	Colleague disturbance	Social well-being
652	displaced and come to here. I and my husband were separated	family loss, push factor	Push factors, Social well-being
653	after we came here. After we came from Nekemt, the child is	family loss	Push factors, Social well-being
703	Respondent: They are very nice	Friend attachment	Social well-being
706	Respondent: Yes, while we are playing something together, I	Friend attachment	Social well-being
707	feel free. Meaning, I am much stressed. Since my current	Friend attachment, stress	Mental well-being, Social well-being
712	Respondent: with individuals who sit here like me or with	Friend attachment	Social well-being
713	individuals there in the rent floor, I feel free when	Friend attachment	Social well-being
714	playing something like that. But I very much stressed when	Friend attachment, stress	Mental well-being, Social well-being
715	I am alone.	Friend attachment, stress	Mental well-being, Social well-being
771	Respondent: we have been together for five years	Family attachment	Social well-being
773	Respondent: Respondent-Ere (??)… (she keeps silent by	colleague conflict	Social well-being
774	nodding her neck). We got a little bit of disagreement and	colleague conflict	Social well-being
775	then we separated	colleague conflict	Social well-being
778	Respondent: Laughter… after this!, no, we can’t.	colleague conflict	Social well-being
781	Respondent: Now the phone is not working, he is not calling	colleague conflict	Social well-being

LineNo	Text	Codes	Synthesis 1
782	and I am not calling too.	colleague conflict	Social well-being
946	fine, but his father is lost and he couldn’t get him.	family loss, modern treatment	Basic need related, Push factors, Soc
948	Respondent: His father is gone. Yes he (the child) didn’t	family loss	Push factors, Social well-being
949	get him, I mean the child’s father. This baby was born here	family loss	Push factors, Social well-being
956	Respondent: Ere (??)…, it is good. They say us, don’t worry	Family attachment	Social well-being
957	something like that. We help each other with friends; we	Family attachment	Social well-being
958	make coffee; have shared eating.	Family attachment	Social well-being
972	Respondent: Ere (??)…, no, we were not arguing.	Family attachment	Social well-being
995	Respondent: Problem…, look, I delivered, I scarify here and	husband loss	Push factors, Social well-being
996	there because of his loss. There is no other attack.	husband loss	Push factors, Social well-being
1000	show ID. We had contract bed. Guys don't touch you in this	Colleague disturbance	Social well-being
1001	country unless you talk to them (??????????). They don’t	Colleague disturbance	Social well-being
1002	touch. This country is good.	Colleague disturbance	Social well-being
1025	(the child’s) father though he (her child’s father) is lost.	family loss	Push factors, Social well-being
1033	Respondent: sends money! Ere… he doesn’t send money. He	husband relation	Social well-being
1034	sent 15, 150 but now he ceased. If he comes, he will manage	husband relation	Social well-being
1035	us. Before this time, his mother is here.	husband relation	Social well-being
1039	Respondent: Ere… you have seen me as I am living outside	loneliness	Social well-being
1040	alone (?? ??? ??? ??? ???? ?????). She went to her son,	loneliness	Social well-being
1041	Addis Ababa. I am living alone (??? ??? ???? ??? ??? ??	loneliness	Social well-being
1042	????? ?? ????)	loneliness	Social well-being
1126	Respondent: He is not considered as he is living. First of	Family attachment, illness	Physical well-being, Social well-being
1127	all, he has a falling disease. When he feels better, he	Family attachment, illness	Physical well-being, Social well-being
1128	comes to the home but when he is sick, he may not come to	Family attachment, illness	Physical well-being, Social well-being
1129	home and spend the night outside. Since he is suffering	Family attachment, illness	Physical well-being, Social well-being
1130	from a mental illness, he is not considered as he is coming	Family attachment, illness	Physical well-being, Social well-being
1131	to home.	Family attachment, illness	Physical well-being, Social well-being
1140	Respondent: When he comes, we eat together, but when he	Family attachment	Social well-being
1141	goes out, he stays outside.	Family attachment	Social well-being
1152	Respondent: nothing, he doesn't work at all. Because he	Family attachment	Social well-being
1153	doesn’t think, with what brain he is thinking? You can	Family attachment	Social well-being
1154	confirm this issue by asking someone else about him. I can	Family attachment	Social well-being
1155	say, he is living with my support.	Family attachment	Social well-being

LineNo	Text	Codes	Synthesis 1
1182	Respondent: Our relationship…, as it is called three stones	Family attachment	Social well-being
1183	are colliding each other (???? ??? ???? ???????), we, who	Family attachment	Social well-being
1184	have child/ren, also arguing now and then immediately we	Family attachment	Social well-being
1185	are playing.	Family attachment	Social well-being
1188	Respondent: Ere…, I'm not arguing with my husband, but with	colleague conflict	Social well-being
1189	mothers in our compound who have a child/ren. Why I fight	colleague conflict	Social well-being
1190	with my husband? because I considered him as he is died. We	colleague conflict	Social well-being
1191	are argued due to our children. A mother may say “your	colleague conflict	Social well-being
1192	child hits my child, why he/she is hitting my child?” like	colleague conflict	Social well-being
1193	this.	colleague conflict	Social well-being
1195	Respondent: We are many. For example, there are two blind	Community attachment	Social well-being
1196	men living together. The rest are married persons.	Community attachment	Social well-being
1199	Respondent: yes, we are drinking coffee together. Our house	Community attachment	Social well-being
1200	owners are Negedyes (Woitos) (????/ ?????), so we don't	Community attachment	Social well-being
1201	drink with them. We drink coffee together with the Amhara	Community attachment	Social well-being
1202	persons.	Community attachment	Social well-being
1475	Answer: Family: I have no mother or father, they are dead,	family loss	Push factors, Social well-being
1476	I have no brother or sister. Only him (her child). I have	family loss	Push factors, Social well-being
1477	no friends. I have nothing. I just fear for my life. The	family loss, fear for life	Emotional well-being, Perception, Pus
1601	daughter, he did not feel well. My husband and relatives do	Family attachment, pregnancy	Social well-being
1602	not know that I am begging. I went to the desert to work	Family attachment, pregnancy, previous work	Social well-being
1607	farming his own and other’s land. But he only helps us	Family attachment, previous work	Social well-being
1608	sometimes. He says “it's only for my daughter. I’m going to	Family attachment, previous work	Social well-being
1609	take my child to my house,” but not sincerely. Before I	Family attachment	Social well-being
1610	came on the street, I worked part-time. But I could not	Family attachment, street life	Social well-being
1615	Answer: Since I was here due to lack of food, I go out in	Community attachment, Friend attachment	Social well-being
1616	the morning and come in at night. There is no relationship	Community attachment, Friend attachment	Social well-being
1617	with others.	Community attachment, Friend attachment	Social well-being
1759	on street. We are five [family size] including me.	family status	Social well-being
1761	Respondent: Yes, he is living with me. But he lives with us	family status, safeguard	Social well-being
1762	to take care of me otherwise he can live on street.	safeguard	Social well-being
1763	Whatever he gets there such as eating leftover he can	safeguard	Social well-being
1764	sustain his life, but he is here with me if someone come to	safeguard	Social well-being
1765	disturb us to protect and take care me. The life here we	brother's living preference, safeguard	Social well-being

LineNo	Text	Codes	Synthesis 1
1797	Respondent: He died long time ago, died when this [little	family loss	Push factors, Social well-being
1798	girl] was a baby; as soon as she was born.	family loss	Push factors, Social well-being
1807	Respondent: I have no connection and I have nothing to	Family attachment	Social well-being
1808	connect with! My family: Only my children and God and this	Family attachment	Social well-being
1809	wall we see [showing their Kenda's house one-side wall	Family attachment	Social well-being
1810	fence that is made of bricks]. This is the most important	Family attachment	Social well-being
1811	thing when the wind blows it protects us, and this is the	Family attachment	Social well-being
1812	reward of God's help.	Family attachment	Social well-being
1814	Respondent: No relationship.	Family attachment	Social well-being
1816	Respondent: Neighbors are supportive to me, they mend when	Community attachment	Social well-being
1817	my tend get torn, tell me not to worry. They are the one	Community attachment	Social well-being
1818	who are holding us while my relative has thrown me out of	Community attachment	Social well-being
1819	the house (Uh). It is the Kebele 14 people who calms and	Community attachment	Social well-being
1820	reach for me when they hear kind of sound since I am left	Community attachment	Social well-being
1821	on street, to hold me they have no the capacity to do that,	Community attachment	Social well-being
1822	this time things are becoming worse.	Community attachment	Social well-being
1825	Respondent: They told me that when it torn advice by saying	Community attachment	Social well-being
1826	do it this way, they gave me that wood, and they gave also	Community attachment	Social well-being
1827	me this, this and that one (pointing to the things that the	Community attachment	Social well-being
1828	neighbor helped them).	Community attachment	Social well-being
1830	Respondent: I don't have anything to say, that is all. . .	Community attachment	Social well-being
1831	I have nothing more to say. I don't have anything that I	Community attachment	Social well-being
1832	mention my dear, this is it, this is it only and I have	Community attachment	Social well-being
1833	nothing to extend…	Community attachment	Social well-being
1836	Respondent: My children have no problem. If I give them,	child relation	Social well-being
1860	They are attached to me to get motherhood affection and	Family attachment	Social well-being
1861	because I love my mother too. Though my father left me, my	Family attachment, family loss	Push factors, Social well-being
1862	mother would be a good person but my mother died. Here they	Family attachment, family loss	Push factors, Social well-being
1863	are too hard to leave their mother. My brother is also	Family attachment	Social well-being
1864	living with me because he found hard to leave me here. But	brother sickness, Family attachment	Physical well-being, Social well-being
1893	Respondent: now she is under our protection, where could	family protection, perceived children's life	Basic need related, Perception, Socia
1894	they go jumping over us?	family protection, perceived children's life	Basic need related, Perception, Socia
1999	for us and thanking this building built by blessed people	Community attachment, hope in God	Social well-being, Spritul well-being
2000	and thanking to the vicinity people.	Community attachment, hope in God	Social well-being, Spritul well-being
2002	Respondent: This one [her daughter], when she sees me	children's desire, children's emotional support	Motivation to leave, Social well-being

LineNo	Text	Codes	Synthesis 1
2003	tearing like now, she says to me “just leave it my mother I	children's desire, children's emotional support	Motivation to leave, Social well-being
2004	will go to America and buy for you a house, don’t worry.”	children's desire, children's emotional support	Motivation to leave, Social well-being
2005	That one [her son] also says “I will carry a sac and you	children's desire, children's emotional support	Motivation to leave, Social well-being
2007	Don’t get tear, instead of losing you we had to work and	children's emotional support	Social well-being
2008	live as much as we can” And if they explain this to me,	children's emotional support	Social well-being
2039	shelter. We get this even because of these blessed	perception, will of neighbours	Perception, Social well-being
2040	villagers and bless the village, bless the vicinity and	will of neighbours	Social well-being
2041	bless the country otherwise who care now? No one cares.	will of neighbours	Social well-being
2081	They[villagers] let me live here since I am a citizen.	Community attachment	Social well-being
2082	Though others bad for you, how could I leave him. So, we	Family attachment	Social well-being
2083	live just by doing that us we can.	Family attachment	Social well-being
2133	But I came begging not to lost my children’s life since I	fear of children loss, husband loss	Emotional well-being, Perception, Pus
2134	lost their father.	fear of children loss, husband loss	Emotional well-being, Perception, Pus
2226	Respondent: We talk on the phone and I don't feel	Family attachment	Social well-being
2227	comfortable going there. You know why I don't feel	Family attachment	Social well-being
2228	comfortable in the countryside.	Family attachment	Social well-being
2230	Respondent: I do not have; I don't know anyone.	Friend attachment	Social well-being
2233	Respondent: Yes, they are available.	Friend attachment	Social well-being

Synthesis 1: Economical well-being

LineNo	Text	Codes	Synthesis 1
	Document: IDI-Translation_Compiled		
33	Respondent: I have nothing. I work here daily and feed them	poorness	Economical well-being, Push fact
93	money, I will pay the credit. Ehh…Even, I don't have metal	child feeding, poorness	Basic need related, Economical
94	pot. I have no money here. I just finished it before I come	poorness	Economical well-being, Push fact
95	here. And some people bring me a pot. Previously, they	material support, poorness	Economical well-being, Push fact
96	brought a small metal pot to me. With that [small metal	material support	Economical well-being
97	pot], I make foods in to pieces, “meferfere” and feed them	child feeding, material support	Basic need related, Economical
194	Respondent: Someone here gives me money. He gave me 200	Financial support	Economical well-being
195	birr and I bought Shiro by 50 birr. I kept 150 birr in the	Financial support	Economical well-being
196	house. Then I bought bread by five birr. I left one	Financial support	Economical well-being
199	and I lost it. I cried and kept quiet. A woman from there	colleague teft, Food support	Basic need related, Economical
200	brought me a fino for children porridge. I fed them	Food support	Basic need related, Economical
201	[children] the night. Then the next day I sat down like	Food support	Basic need related, Economical
271	The district then gave me two sheets of nightgown, a	clothes support, Food support	Basic need related, Economical
272	military coat and ten kilos of fino, and I sold that fino	clothes support	Economical well-being
273	and bought sorghum. I mean, because sorghum is high in	Food support	Basic need related, Economical
318	and when we did not find it, we did not eat. Ehh…, someone	food shortage, Food support, perceived severity	Basic need related, Economical
338	Respondent: Some rich people dressed the them [children],	clothes support	Economical well-being
339	and they [rich people] go. We are wait up three o'clock in	clothes support	Economical well-being
340	the evening. Some people come and buy macaroni, something	Food support	Basic need related, Economical
341	to me, and go. I cook that, and I give it to them by	Food support	Basic need related, Economical
342	myself, but my internal [stomach] is empty. We are fasting,	food shortage, Food support	Basic need related, Economical
343	we have nothing to do. “Mariamn.”	Food support	Basic need related, Economical
349	Respondent: When I cry to the rich person who pass here, he	Supporting childcare	Economical well-being
350	will not leave me alone and he will take care of them. If	Supporting childcare	Economical well-being
351	they are sick, he will take care of them, and he will never	Supporting childcare	Economical well-being
352	leave.	Supporting childcare	Economical well-being
390	house, I think what I should pay? For example, we go to a	Financial support, forgetfullness	Economical well-being, Mental w
391	store on Friday and ask money for rent, and they give us	Financial support	Economical well-being
392	money for a rent.	Financial support	Economical well-being
432	Respondent: Our problem in the street is hunger. Even after	Food support, poorness	Basic need related, Economical
433	feeding the children, I still spend the night by fasting.	Food support	Basic need related, Economical
434	I'm telling you the truth. Why is the coin disappearing?	Food support	Basic need related, Economical

LineNo	Text	Codes	Synthesis 1
474	Respondent: 70 Birr for food. If we do not do much, we will	Income source	Economical well-being
475	make fifty birr.	Income source	Economical well-being
663	Respondent: I collected what they give me here, if I have;	Financial support	Economical well-being
667	Respondent: I may get 60, or 70Birr and sometimes I can't	Financial support	Economical well-being
668	get money.	Financial support	Economical well-being
683	Respondent: We brought some clothes from there, without	clothes support	Economical well-being
684	which, here is nothing.	clothes support, lack of support	Economical well-being
726	Respondent: the woman [the owner of the rented house] gave	material support	Economical well-being
727	us a pot. If I get something to be cooked, I take and make	child feeding, material support	Basic need related, Economical
733	Respondent: I am buying a jar (jerry-can) of water for one	Hygiene related economy	Economical well-being
734	birr and I use it. I am using that jar of water carefully	Hygiene related economy	Economical well-being
735	not to frequently buy or pay another one birr.	Hygiene related economy	Economical well-being
738	Respondent: still I have nothing but neighbors are giving	material support	Economical well-being
739	me some things.	material support	Economical well-being
767	everything necessary for the family. Now, since I have no	poorness, support	Economical well-being, Push fact
768	money to full fill the necessary things, I live in trust of	hope in God, poorness	Economical well-being, Push fact
798	for us". Even if you enter in to their home to work, you	Financial support	Economical well-being
799	will not be paid well. For 200 or 300birr!, I don't know	Financial support, perception	Economical well-being, Percepti
917	Respondent: Food! as you were seeing us, we sit with other	Food support	Basic need related, Economical
918	beggars, while they are eating daily bolus of food (????	Food support	Basic need related, Economical
919	??? ????), we are asking them to eat.	Food support	Basic need related, Economical
922	Respondent: Ere (??)…the money is much	Financial support	Economical well-being
924	Respondent: I can get 30biir, 40birr, on average 20birr	Financial support	Economical well-being
925	something like that.	Financial support	Economical well-being
1083	and sometimes I can get also 100birr. The money I get	Financial support	Economical well-being
1084	varies from day to day because I don't spend full time on	Financial support, perception	Economical well-being, Percepti
1085	the street with the reason of my children i.e. I don't	Financial support, perception	Economical well-being, Percepti
1276	the small kid regarding fire. She will feed the small kid	clothes support, desire to leave, support from child	Economical well-being, Motivatio
1338	somebody takes me, I can go to that area. After I came from	Financial support, support	Economical well-being
1339	Gojeb, I have got only 2,000 birr from the government at	Financial support	Economical well-being

LineNo	Text	Codes	Synthesis 1
1340	the time of Corona virus. I did not have an ID, fortunately	Financial support	Economical well-being
1341	they registered me together with the neighborhood	Financial support	Economical well-being
1342	individuals and they handed me a back book. It means I	Financial support	Economical well-being
1343	received the money from a commercial bank. First, they were	Financial support	Economical well-being
1344	informed us that we can get help every 6 months but after	Financial support	Economical well-being
1407	Answer: No, that's all.	Income source	Economical well-being
1410	Someone gave us a blanket (koborta) and we are wearing it	material support	Economical well-being
1411	while we are sleeping.	material support	Economical well-being
1438	Answer: We eat what we get. It was given from other. Or we	food shortage, Food support	Basic need related, Economical
1439	can buy bread and eat by the money we get from begging.	Food support	Basic need related, Economical
1478	people living with me there (Abuye Tsebel with) are praying.	material support	Economical well-being
1479	They give us mattress and they inform me baptize the baby.	material support	Economical well-being
1501	There is insult: Some say why do not you work and some	Financial support, Insult	Economical well-being, Psycholo
1502	give us birr.	Financial support, Insult	Economical well-being, Psycholo
1586	Answer: I'm afraid of my child being stolen since she	child protection, poorness	Basic need related, Economical
1587	approaches with others by calling my father and my mother.	child protection, poorness	Basic need related, Economical
1588	I'm afraid she'll be crashed by a car. There is no money	child protection, modern treatment, poorness	Basic need related, Economical
1672	Respondent: I am working dry waste collection. I just get	Income source	Economical well-being
1673	two thousand birrs only; you buy injera, you shero and	low income for livelihood	Economical well-being
1674	berbere, what it could do? (Long pause) you buy firewood,	low income for livelihood	Economical well-being
1675	then after unable to cover house rent and I ended up on	low income for livelihood	Economical well-being
1676	street, what other option do I have except this?	low income for livelihood	Economical well-being
1679	Respondent: This is the only one, no other	Income source	Economical well-being
1681	Respondent: It is only two thousand per month. Two thousand	low income for livelihood	Economical well-being
1682	birr; to feed us, to do something else, you pay from it for	low income for livelihood	Economical well-being
1683	children schooling, I have nothing to hide from it. Nothing	low income for livelihood	Economical well-being
1684	else and the children are also students.	child education, low income for livelihood	Basic need related, Economical
1733	Respondent: What does two thousand birr can do?	low income for livelihood	Economical well-being
1804	Respondent: Yes, I have nothing that I own.	poorness	Economical well-being, Push fact
1866	eat. We used to eat this [monthly salary] just for five	brother sickness, Income source	Economical well-being, Physical
1867	family; only two thousand birr. It [the salary] is all what	Income source	Economical well-being
1868	we used to live; it is what we eat, it is water to drink,	Income source	Economical well-being
1869	it injera to eat, it is charcoal for cooking. You see, how	christian life, Income source	Economical well-being, Spritul w

LineNo	Text	Codes	Synthesis 1
1974	if he sells fattened cattle, he can be a trader. But leave	desire to work, poorness	Economical well-being, Motivatio
1975	alone affording that I am homeless and I let them sleep on	desire to work, poorness	Economical well-being, Motivatio
1976	the ground. How can they now? Either we don't have	poorness	Economical well-being, Push fact
1977	something to say this way, everybody has way-outs but we	poorness	Economical well-being, Push fact
1978	have no way-out. Either I don't have a rural land or I	poorness	Economical well-being, Push fact
1979	don't own any way-outs when I am here also. I lost every	poorness	Economical well-being, Push fact
1980	way-outs and I sat down asking him for way-outs. Even, you	poorness, poors not heard	Economical well-being, Percepti
1984	me. And I can't do anything. I wish God to bring a	hope in God, poorness, poors not heard	Economical well-being, Percepti
1996	sell near to house and take care of the children. But I	desire to work, poorness	Economical well-being, Motivatio
1997	have no capacity to do all this, both the day and the night	poorness	Economical well-being, Push fact
1998	are dark for us. We are here asking God to do better thing	hope in God, poorness	Economical well-being, Push fact
2010	conscience wound for them. I have incapable of doing	hopelessness, poorness	Economical well-being, Emotion
2011	anything. But practically nothing can be done. It is	hopelessness, poorness	Economical well-being, Emotion
2012	impractical if I say I will do this and this to you because	poorness	Economical well-being, Push fact
2099	Respondent: What can I describe? It is what I get with	Income source, perception	Economical well-being, Percepti
2100	suffering and begging daily. From what I get from people;	Income source, perception	Economical well-being, Percepti
2101	half is for child, and half is also for family. Nothing I	Income source, perception	Economical well-being, Percepti
2102	can do more than this.	Income source, perception	Economical well-being, Percepti
2105	Respondent: I didn't buy anything from the market, I buy	poorness	Economical well-being, Push fact
2106	Shiro, only Shiro that I buy and eat. I buy Injera.	poorness	Economical well-being, Push fact
2109	Respondent: It is from what I earned here.	Income source	Economical well-being
2112	Respondent: Yes, nothing else. Those who feel empathy gives	Income source	Economical well-being
2113	me, who didn't feel empathy will leave me. And other go	Income source	Economical well-being
2114	feeling empathy. Now, Shiro has become more expensive. It	Market Inflation	Economical well-being
2115	became very difficult.	Market Inflation	Economical well-being
2119	Respondent: Ah! What do I get? Where do I get it from? So,	Market Inflation	Economical well-being
2124	Respondent: Yes, that's what I have to spend for food and	Income source	Economical well-being
2125	house rent.	Income source	Economical well-being

Synthesis 1: Mental well-being

LineNo	Text	Codes	Synthesis 1
	Document: IDI-Translation_Compiled		
119	urine smells bad. And like that it is changing, I'm worried	pusy urine, Worry	Mental well-being, Physical well-being
120	about. Whether this is the strength of my illness or not.	Worry	Mental well-being
121	When I get stressed, my eyes change. It looks like red. I'm	eye redness, stress	Mental well-being, Physical well-being
122	stressed. I don't sleep on my left side. When I was about	sleeping disturbance, stress, troubling pain	Mental well-being, Physical well-being
179	I have never felt so overwhelmed. I'm worried. I am always	Worry	Mental well-being
180	pregnant. When they went there, I said, "Stop!" When they	Worry	Mental well-being
387	Respondent: First of all, when I think of rent, I have a	forgetfullness	Mental well-being
388	time that I will forget. I forget why because the house	forgetfullness	Mental well-being
389	renter here doesn't pass a month. When I remember rented	forgetfullness	Mental well-being
390	house, I think what I should pay? For example, we go to a	Financial support, forgetfullness	Economical well-being, Mental well-be
557	by yourself" Then anxiety will come. There is a woman. She	anxiety	Mental well-being
560	come by stealing." At that moment, our head hurts.	anxiety, blaming	Mental well-being, Psychological abus
659	that time, I become disappointed, but since you have no	Insult, stress	Mental well-being, Psychological abus
660	choice to work, you keep silent because you do nothing.	Insult, stress	Mental well-being, Psychological abus
707	feel free. Meaning, I am much stressed. Since my current	Friend attachment, stress	Mental well-being, Social well-being
708	and previous status is not related, I am much stressed. But	stress	Mental well-being
709	when we play like this, the thing that stresses my brain	stress	Mental well-being
710	will be removed. But other is nothing.	stress	Mental well-being
714	playing something like that. But I very much stressed when	Friend attachment, stress	Mental well-being, Social well-being
715	I am alone.	Friend attachment, stress	Mental well-being, Social well-being
743	Respondent: I get very stressed when I think of sitting	stress	Mental well-being
744	alone, but while I am plying about that thing together with	stress	Mental well-being
745	my friends (individuals like me) or with floor renters, I	stress	Mental well-being
746	find it easier. I'm very worried about where I used to be	stress, Worry	Mental well-being
747	(status of me when in my house) and where I am now, but it	Worry	Mental well-being
748	makes it easier for me to worry when I play like this.	stress, Worry	Mental well-being
765	Previously, when I was in my house [in Lekemt], I was not	Worry	Mental well-being
766	worried about the cost, my husband was fulfilling	support, Worry	Mental well-being
986	Respondent: I do not worry about anything. That is, going	Worry	Mental well-being
987	and seeing my children is enough. They are in a rural area.	Worry	Mental well-being
988	I will go frequently to see them.	Worry	Mental well-being

LineNo	Text	Codes	Synthesis 1
1445	Answer: My body is fine. But I have sometimes stressed.	stress	Mental well-being
1447	Answer: Yes. I'm so angry about since my childbirth	stress	Mental well-being
1449	Answer: It's going to be a year. After I give birth to the	stress	Mental well-being
1450	child, he[her child] is now a year old.	stress	Mental well-being
1487	Answer: Sometimes I cannot do work. I become depress. I	depress	Mental well-being
1488	can't do work.	depress	Mental well-being
1620	Answer: lack of sleeping, insomnia, anxiety, I think a lot	anxiety, insominia, stress	Mental well-being
1621	(tearing)	insominia, stress	Mental well-being
1735	Respondent: Nothing will happen, I kill myself if the	suicidal ideation	Mental well-being
1736	government just takeover my children…(Interrupted by tears)	suicidal ideation	Mental well-being
1789	Respondent: They are all healthy but now they are probably	Worry children's mental health	Mental well-being
1790	unhealthy. And their mental is getting tired of	children's worry, Worry children's mental health	Emotional well-being, Mental well-bein
1791	overthinking. On top of this, … They think that this all	children's worry	Emotional well-being, Mental well-bein
1792	are happening to them because of the death of their father	children's worry	Emotional well-being, Mental well-bein
1793	and their mother being alone. They started to tear and to	children's worry	Emotional well-being, Mental well-bein
1794	talk alone. The next is being mad, (the respondent was in	children's worry, Worry children's mental health	Emotional well-being, Mental well-bein
1795	grief mood), they are healthy other than this.	Worry children's mental health	Mental well-being
1852	otherwise. They just sit here and do their daily homework	children's worry, suggest change living place	Emotional well-being, Mental well-bein
1853	saying “what are we going to eat there?” and “if we live	children's worry	Emotional well-being, Mental well-bein
1854	here, this is not enough for living?”, this is all.	children's worry	Emotional well-being, Mental well-bein
1856	Respondent: Yes, they worry. Hmmm…they get pity for what	children's worry	Emotional well-being, Mental well-bein
1857	they can't solve! That is, it is better to work than to	children's worry	Emotional well-being, Mental well-bein
1858	think. Nowadays, we see a lot of people going crazy and go	children's worry, Worry children's mental health	Emotional well-being, Mental well-bein
1859	mad because of overthinking. The mother has a lot of vision.	Worry children's mental health	Mental well-being
2249	Respondent: I'm facing that! Baking is a problem, shopping	Worry	Mental well-being
2250	is a problem, what to buy, what to bake, I have so much	Worry	Mental well-being
2251	trouble!	Worry	Mental well-being
2254	Respondent: When I left that stuff, I just remembered my	stress	Mental well-being
2255	stuff, I remembered everything, it was just so much stress.	stress	Mental well-being
2256	It causes stress why not it causes stress?!	stress	Mental well-being
2258	Respondent: Yes; What can I do about it once I lose it?	stress	Mental well-being
2259	[with condolence feeling]	stress	Mental well-being

Synthesis 1: Emotional well-being

LineNo	Text	Codes	Synthesis 1
	Document: IDI-Translation_Compiled		
40	willing, I was using that, because when the cars went, and	fear of accident, hope in God	Emotional well-being,
41	returned, I suspected they crushed them [children]. It's a	fear of accident, leg stiffness	Emotional well-being,
50	mother or a father. What can I do to help children? I gave	family loss, hopelessness	Emotional well-being,
51	the older child, he is a 6-year-old, to person. Someone	child beat, hopelessness	Emotional well-being,
176	my immature children are here in the road who are exposed	fear of accident	Emotional well-being,
177	to road traffic accident when they are paying in the road	fear of accident	Emotional well-being,
178	in the whole 24 hours.	fear of accident	Emotional well-being,
181	[cares] got up and came back, I do not have a leg run and	fear of accident	Emotional well-being,
182	bring them, I just always suspect that they crush them.	fear of accident	Emotional well-being,
224	going up. When a car is coming down, car is coming up, I	fear of accident, perception	Emotional well-being,
225	suspect it crushes them. I don't want to sit on the street	desire to leave, fear of accident	Emotional well-being,
1144	Respondent: paying house rent…, if his own safety is fine	fear of accident	Emotional well-being,
1145	(?? ??? ???… ?? ??? ???? ??? ???). My usual concern is that	fear of accident	Emotional well-being,
1146	one day he may get into a car and sustain accidents. This	fear of accident	Emotional well-being,
1147	kind of disease doesn't say today and tomorrow (doesn't	fear of accident	Emotional well-being,
1148	give time). First, if he may find water and will drown	fear of accident	Emotional well-being,
1149	into it or he may get fire and will fall into the fire.	fear of accident	Emotional well-being,
1452	Answer: the reason is due to being angry due to my child	anger	Emotional well-being
1453	birth	anger	Emotional well-being
1455	Answer: The reason is that I gave birth. I hated having a	anger, hate child	Emotional well-being
1456	child without money.	anger, hate child	Emotional well-being
1477	no friends. I have nothing. I just fear for my life. The	family loss, fear for life	Emotional well-being,
1490	Answer: I'm very angry. But, I can do nothing by doing so,	anger	Emotional well-being
1491	and nowhere to go. I don't like sleeping on the porch like	anger, hopelessness	Emotional well-being
1512	up for me, if no, I can't do anything to cure from the	child sickness, hope in God, hopelessness	Basic need related, E
1513	illness.	child sickness, hope in God, hopelessness	Basic need related, E
1527	Answer: What can I take with me? What can I eat? What	hopelessness, perception	Emotional well-being,
1528	shall I go out to eat? But when I am in trouble, I kept	hopelessness, perception	Emotional well-being,
1529	silent; and continue this street life.	hopelessness, perception	Emotional well-being,

LineNo	Text	Codes	Synthesis 1
1629	her head down).	Hit, raped, tearing, theft	Emotional well-being,
1632	Answer: She just cried.	tearing	Emotional well-being
1635	She just cried.	tearing	Emotional well-being
1718	Respondent: Oh, it is very difficult my dear! It is very	Feels sad, perception	Emotional well-being,
1719	difficult very difficult (Sadness on their faces)	Feels sad, perception	Emotional well-being,
1721	Respondent: As you are here, the madman comes, the wind	perception, tearing	Emotional well-being,
1722	comes, and beast comes, when I takeoff this (pointing	perception, tearing	Emotional well-being,
1723	towards the plastic) the rain gets the children. So, it is	perception, tearing	Emotional well-being,
1724	very difficult (tear is coming in her eyes)…(shedding tears	perception, tearing	Emotional well-being,
1725	on her cheeks)…	perception, tearing	Emotional well-being,
1777	is the word of God gives which gives me the strength. The	family/stepmother's push, Feels sad, Praying	Emotional well-being,
1778	things that make me teared and saddened are when I see my	family/stepmother's push, Feels sad	Emotional well-being,
1779	children here sheltered at me and they [stepmother] left us	family/stepmother's push, Feels sad	Emotional well-being,
1780	in an open field. Ehh, ehh…when it gets rain, they wear a	child sufferring, family/stepmother's push, Feels sad	Basic need related, E
1790	unhealthy. And their mental is getting tired of	children's worry, Worry children's mental health	Emotional well-being,
1791	overthinking. On top of this, … They think that this all	children's worry	Emotional well-being,
1792	are happening to them because of the death of their father	children's worry	Emotional well-being,
1793	and their mother being alone. They started to tear and to	children's worry	Emotional well-being,
1794	talk alone. The next is being mad, (the respondent was in	children's worry, Worry children's mental health	Emotional well-being,
1852	otherwise. They just sit here and do their daily homework	children's worry, suggest change living place	Emotional well-being,
1853	saying “what are we going to eat there?” and “if we live	children's worry	Emotional well-being,
1854	here, this is not enough for living?”, this is all.	children's worry	Emotional well-being,
1856	Respondent: Yes, they worry. Hmmm…they get pity for what	children's worry	Emotional well-being,
1857	they can't solve! That is, it is better to work than to	children's worry	Emotional well-being,
1858	think. Nowadays, we see a lot of people going crazy and go	children's worry, Worry children's mental health	Emotional well-being,
1902	and want to rape, but this times it is not AIDS that the	fear of geting disease, protection from children, rape intention	Emotional well-being,
1903	only disease, there are many patients with the other	fear of geting disease, rape intention	Emotional well-being,
1904	disease. For example, now I feeling warm since you are here	fear of geting disease, perceived risks	Emotional well-being,
1948	citizen now, I will be sent by a car to my own country. The	Feeling, perception, poors not heard	Emotional well-being,
1949	days are against and the hours (everything) are against me,	Feeling, perception	Emotional well-being,
1950	otherwise no one deserves this live leave alone a person	Feeling, perception	Emotional well-being,
1951	like me who have children; they are the one who takeover	Feeling, investing on children, perception	Emotional well-being,
1971	thank you. But where does all this come from? I have no	hopelessness	Emotional well-being
1972	money to let them trade, ehhh…, I have no a place to let	hopelessness	Emotional well-being

LineNo	Text	Codes	Synthesis 1
1973	them work there. Today, ehhh, if he trades chickens, ehhh,	desire to work, hopelessness	Emotional well-being,
2010	conscience wound for them. I have incapable of doing	hopelessness, poorness	Economical well-being
2011	anything. But practically nothing can be done. It is	hopelessness, poorness	Economical well-being
2130	[Jimma] than the life here. But I left saddened there	fear of children loss, perception	Emotional well-being,
2131	[Jimma] and I came up with the idea that these people would	fear of children loss, perception	Emotional well-being,
2132	destroy my children. Otherwise, that one was better for me.	fear of children loss, perception	Emotional well-being,
2133	But I came begging not to lost my children's life since I	fear of children loss, husband loss	Emotional well-being,
2134	lost their father.	fear of children loss, husband loss	Emotional well-being,

Synthesis 1: Spritul well-being

LineNo	Text	Codes	Synthesis 1
	Document: IDI-Translation_Compiled		
26	years ago. As it started, I went to Gishen DebreKerbe	Illness duration, swelling, traditional treatment	Physical well-being, Spritul w
27	Tsebel. And also, I went to Michael and Abunehara tsebel.	traditional treatment	Spritul well-being
38	me a lot of leftovers. Ehh…., let me tell you, I've been	child feeding, traditional treatment	Basic need related, Spritul we
39	drinking ten liters Tsebel a day and I'm too emaciated. God	Emaciated, hope in God, traditional treatment	Physical well-being, Spritul w
40	willing, I was using that, because when the cars went, and	fear of accident, hope in God	Emotional well-being, Percept
103	area], thanking God. I am not going to anywhere at night.	Thanking God	Spritul well-being
164	cave for eight months and was baptized. I was baptized for	attending holywater	Spritul well-being
165	eight months, eating only whole grains. When I was baptized,	attending holywater	Spritul well-being
166	the woman next to me baptizing her girl was giving me the	attending holywater	Spritul well-being
167	grain. There, I would fall asleep, thinking it was magic. I	attending holywater	Spritul well-being
168	did not hold the baby himself. It hurts me: It hurts a lot	attending holywater	Spritul well-being
169	me; it hides me; It crushes me. Instead of this, I could	attending holywater	Spritul well-being
170	not confuse. And when I went Mariam, When I went Gishen,	attending holywater	Spritul well-being
171	when I went Immanuel, and I went to a lot of Tsebel areas,	attending holywater	Spritul well-being
172	I did not face mind confusion.	attending holywater	Spritul well-being
186	more mature than them, I gave her to someone else. Michael	Effort to end street, Praying	Efforts to leave, Spritul well-b
187	knows, I am always crying in front of Michael get way. Ehh…,	Praying	Spritul well-being
188	I've went and drop "selet" to every church, even Amanual.	Praying, Selet	Spritul well-being
231	and fall of a church, a monastery with a little child.	Joining monastries	Motivation to leave, Spritul w
232	Where will I go forever in handicap? If there is a better	Joining monastries	Motivation to leave, Spritul w
233	monastery, I will take Meat Blood (siga demun), but the	siga demu	Spritul well-being
234	children are immature. I now have a repentant father. I	religious support, siga demu	Spritul well-being
235	said to him I did not do nothing. So, I asked him to give	religious support	Spritul well-being
236	me Meat, Blood, "siga demun". He said "they are children;	religious support	Spritul well-being
237	They could mature for you. Unless they will ask you in	religious support	Spritul well-being
238	heaven. Where do you go as you spread them like grain in	religious support	Spritul well-being
239	the street go? If you hurt your health, their health will	religious support	Spritul well-being
240	hurt too. Even other persons raise children at home, they	religious support	Spritul well-being
241	beg to raise their child. Please calm down. At least until	religious support	Spritul well-being
242	they reach ten years" he said. Once again, I was disturbed.	religious support	Spritul well-being
266	so far no one was able to support me. I went to Libokemkem	attending holywater, lack of support	Spritul well-being
267	woreda for tsebel, I stopped consuming food. Female's	attending holywater, food shortage	Basic need related, Spritul we
346	treatment. Who else can help me? I believe in God. Who will	hope in God, lack of support	Spritul well-being

LineNo	Text	Codes	Synthesis 1
404	say Lord George gives me a solution for this. It's true	hope in God, perception	Perception, Spritul well-being
768	money to full fill the necessary things, I live in trust of	hope in God, poorness	Economical well-being, Push
769	God but not in myself.	hope in God	Spritul well-being
801	to pass this day like this, to rare/grows up/ my child. If	hope in God, perception	Perception, Spritul well-being
802	God blessed me, he can pass this horrific life. If God did	hope in God	Spritul well-being
803	not welcome me, he, himself knows. They insult me something	hope in God, Insult	Psychological abuse, Spritul
817	Respondent: ????? ??… if it is God's willing and I get	God will	Spritul well-being
818	something, I will leave street life otherwise no other	God will	Spritul well-being
824	day work and expensing the money for what you want and	hope in God	Spritul well-being
825	living by saying thanks to God. Like this, sleeping with	hope in God, perception	Perception, Spritul well-being
834	if God is willing, it becomes bigger. A little work is	Desire to make money, hope in God, perception	Motivation to leave, Perceptio
847	Respondent: No one can help me. Like this suddenly, who	hope in God, lack of support	Spritul well-being
848	knows, there is God. God…who knows, suddenly may help me.	hope in God	Spritul well-being
941	now thanks to God, I'm still fine. I have malaria and I	Health care, Thanking God	Basic need related, Spritul we
1028	Respondent: Ere… I didn't think ever, thanks to God. Up to	Effort to end street, Thanking God	Efforts to leave, Spritul well-b
1077	before this time. Thanks to God, I am fine till now.	no health problem, Thanking God	Physical well-being, Spritul w
1110	Respondent: Praise to God for clothes, even, no problem	child feeding, Thanking God	Basic need related, Spritul we
1150	This is the help of God but not the help of him.	God will	Spritul well-being
1174	Respondent: Ere…, I'm fine, thanks to God. There is some	no health problem, Thanking God	Physical well-being, Spritul w
1175	problem on my eye; otherwise, I'm fine, thanks to God. One	illness, Thanking God	Physical well-being, Spritul w
1214	Respondent: Ere…, my mother Kidanemihret (??… ??? ???????),	traditional treatment	Spritul well-being
1215	I haven't tried it (?? ????? ????). I have no such a habit	traditional treatment	Spritul well-being
1216	except Dirsane Michael (???? ????). When they are sick, I	modern treatment, traditional treatment	Basic need related, Spritul we
1220	Respondent: I buy it from the sellers located on the	traditional treatment	Spritul well-being
1221	roadside and tie on their neck like this (she is pointing	traditional treatment	Spritul well-being
1222	the tied thing on her elder child neck). Except this, even	traditional treatment	Spritul well-being
1223	I haven't tried to tie an evil (??) medicine with the	traditional treatment	Spritul well-being
1224	reason of the God keeps them. Medehanialm you are! (???????	hope in God, traditional treatment	Spritul well-being
1225	??? ???), You know what you gave me (?????? ??? ????? ??),	hope in God	Spritul well-being
1226	because he gave them to me and he took them while he want	hope in God	Spritul well-being
1227	(?????? ???? ?? ?? ???? ?? ??).	hope in God	Spritul well-being

LineNo	Text	Codes	Synthesis 1
1237	sit together with anyone. Thanks to God, I fine up to now.	no attack, Thanking God	Physical abuse, Spritul well-b
1248	Respondent: first of all Greetings!, then where are we go?	Praying	Spritul well-being
1322	Respondent: I always beg to my God. During sleep time, I	Praying	Spritul well-being
1323	always tell him (God) that please give me what you have	Praying	Spritul well-being
1324	decided for me and then I can take my own choice. Always,	Praying	Spritul well-being
1325	while I am sleeping, I am informing my God to quit from	Praying	Spritul well-being
1326	this type of work. Now, I and he are knew how he gives it	Praying	Spritul well-being
1327	for me (???? ???? ?????? ??? ?? ?? ?? ??????). Unwise man	perception, Praying	Perception, Spritul well-being
1329	disgusting thing. "In the name of the Father, and of the	perception, Praying	Perception, Spritul well-being
1330	Son, and of the Holy Spirit…"	Praying	Spritul well-being
1345	they offered 200birrr, they have stopped it. I have no	hope in God	Spritul well-being
1346	helper except this sky.	hope in God	Spritul well-being
1348	Respondent: My helpers are only persons of Bahir Dar and my	hope in God, support	Spritul well-being
1349	God..	hope in God	Spritul well-being
1421	care. There is no care in the dust. There is no washing. It	hope in God, perception	Perception, Spritul well-being
1422	is God who holds us accountable. It is by God's will.	hope in God	Spritul well-being
1436	Answer: I have never been sick, thank you my God.	Thanking God	Spritul well-being
1512	up for me, if no, I can't do anything to cure from the	child sickness, hope in God, hopelessness	Basic need related, Emotiona
1513	illness.	child sickness, hope in God, hopelessness	Basic need related, Emotiona
1590	couldn't afford for the care. I took my child to holy water,	modern treatment, traditional treatment	Basic need related, Spritul we
1591	but they asked me to pay money for it. Due to that I left	modern treatment, traditional treatment	Basic need related, Spritul we
1592	the holy water. So, I could not baptize. My daughter is 3	modern treatment, traditional treatment	Basic need related, Spritul we
1593	years old.	modern treatment, traditional treatment	Basic need related, Spritul we
1774	signaling hopelessness). So far, I survived here because of	ART user, God will	Physical well-being, Spritul w
1775	the will of God. I woke up at night and say this is a	God will, Praying	Spritul well-being
1776	challenge what the God present for whom he loves them, this	Praying	Spritul well-being
1777	is the word of God gives which gives me the strength. The	family/stepmother's push, Feels sad, Praying	Emotional well-being, Push fa
1869	it injera to eat, it is charcoal for cooking. You see, how	christian life, Income source	Economical well-being, Spritu
1870	can a Christian be here? I got this also because these	christian life, neighbour's support	Spritul well-being
1984	me. And I can't do anything. I wish God to bring a	hope in God, poorness, poors not heard	Economical well-being, Perce
1985	better/blessed day for us and create a solution for my	hope in God	Spritul well-being
1986	children. Then, they just get a place to live and work and	hope in God	Spritul well-being

LineNo	Text	Codes	Synthesis 1
1987	feed themselves, what can I say to other this? Now this	desire to work, hope in God	Motivation to leave, Spritul w
1998	are dark for us. We are here asking God to do better thing	hope in God, poorness	Economical well-being, Push
1999	for us and thanking this building built by blessed people	Community attachment, hope in God	Social well-being, Spritul well-
2000	and thanking to the vicinity people.	Community attachment, hope in God	Social well-being, Spritul well-
2013	I have nothing. Mainly the creator who we have, who created	hope in God	Spritul well-being
2014	us, nothing other than this.	hope in God	Spritul well-being
2160	compared with assets. These children; first this child's	child disability, God will, Value life than assets	Physical well-being, Spritul w
2161	[little child] disability that God make him lost his body.	child disability, God will	Physical well-being, Spritul w
2163	came and so far, I am trying to survive, thanks to him.	push factor, Thanking God	Push factors, Spritul well-bein
2181	position and walk/go. Thank God for another problem.	child disability, desire for education, Thanking God	Motivation to leave, Physical

Synthesis 1: Perception

LineNo	Text	Codes	Synthesis 1
	Document: IDI-Translation_Compiled		
40	willing, I was using that, because when the cars went, and	fear of accident, hope in God	Emotional well-bei
41	returned, I suspected they crushed them [children]. It's a	fear of accident, leg stiffness	Emotional well-bei
70	(she is pointing the waist), I look like normal. But it is	mobility difficulty, societal perception	Perception, Physic
80	Respondent: When he is hungry, he goes to the fish house.	child missbehave	Perception
81	Right there, “keahune keahune man setegn eyeale, ayen ayen	child missbehave	Perception
82	new yemeayew.” (??? ???? ??? ?? ??? ??? ??? ??? ?? ?????).	child missbehave	Perception
83	He sees what they are eating. If I get school, I plan to	child missbehave	Perception
158	Respondent: I have encountered nothing on the street. I on	perception	Perception
159	the street, thank you [God], those who have drop, but those	perception	Perception
160	who haven’t have, passed simply. I have experienced	perception	Perception
161	nothing.	perception	Perception
175	one. Ehh, just I'm falling on the street at this age. Ehh,	loneliness, perception	Perception, Social
176	my immature children are here in the road who are exposed	fear of accident	Emotional well-bei
177	to road traffic accident when they are paying in the road	fear of accident	Emotional well-bei
178	in the whole 24 hours.	fear of accident	Emotional well-bei
181	[cares] got up and came back, I do not have a leg run and	fear of accident	Emotional well-bei
182	bring them, I just always suspect that they crush them.	fear of accident	Emotional well-bei
183	They don't know when you tell them since they are babies.	Need adult protection	Perception
184	Because they do not know the bad and good things; two	Need adult protection	Perception
185	children walking around. The that child [oldest child] is	Effort to end street, Need adult protection	Efforts to leave, P
222	where so many things are being thrown. Spread the children	perception	Perception
223	here that I can't find them running anywhere. My soul is	perception	Perception
224	going up. When a car is coming down, car is coming up, I	fear of accident, perception	Emotional well-bei
225	suspect it crushes them. I don't want to sit on the street	desire to leave, fear of accident	Emotional well-bei
243	Above my waist I am normal (she is pointing the waist),	societal perception	Perception
244	there were many people on the street, and they looked at me	societal perception	Perception
245	in amazement while I beg.	societal perception	Perception
304	Respondent: I just don't want to be on the street. I want	desire to leave, perception	Motivation to leave
305	to go out looking for a daily labor	desire to leave, perception	Motivation to leave
307	Respondent: It's a matter of loss. Living on the street is	perception	Perception
308	not life. For me, is not a job. Now, for example, we are	perception	Perception
309	looking at the face of others whether the give something to	perception	Perception

LineNo	Text	Codes	Synthesis 1
310	us or not. We have no choice, with children, where we go?,	perception	Perception
311	but I in my own, I was so overwhelmed. Why because sitting	desire to leave, perception	Motivation to leave
312	here and selling corn is an alternative work. I don't want	desire to leave, perception	Motivation to leave
313	to beg, but it is a matter of losing to do this. I prefer	desire to leave, perception	Motivation to leave
314	this (selling corn), I don't want the street.	desire to leave, perception	Motivation to leave
316	Respondent: We have a time without food in the night. There	food shortage, perceived severity	Basic need related
317	is a time we do not have injera. We eat when we find it,	food shortage, perceived severity	Basic need related
318	and when we did not find it, we did not eat. Ehh…, someone	food shortage, Food support, perceived severity	Basic need related
319	who pass to here buys lunch or breakfast for us, and goes.	food shortage, perceived severity	Basic need related
320	Ehh…, we spend most of our time without eating and drinking,	food shortage, perceived severity	Basic need related
321	because from where and what we eat?	food shortage, perceived severity	Basic need related
324	Respondent: I'm not happy. I don't like living on this	perception	Perception
325	street. We are here up to this day due to lack of man.	lack of support, perception	Perception
327	Respondent: Why do you think if I sleep here with children?	perception, sleeping place	Basic need related
328	It is so cold. So, I rented the dormitory by more than 500	perception, sleeping place	Basic need related
334	I grow up these [the two children] by begging to the best	perception	Perception
335	of my ability.	perception	Perception
371	Respondent: We left on the street due to poorness, the	perception	Perception
372	people blame us by saying you are from beggar generation.	blaming, perception	Perception, Psych
373	It's a matter of losing, not preference. They throw in ten	perception	Perception
374	cent, and they say “see her, why not she does to eat?” If	perception	Perception
403	Respondent: Street life is too bitter to me. All the day I	perception	Perception
404	say Lord George gives me a solution for this. It's true	hope in God, perception	Perception, Spritul
405	because I'm so angry. It is disgusting to walk around the	perception	Perception
406	church and any place repeatedly in the morning. Not only	perception	Perception
407	here. If I did not get coin here, I traveled up to Adit and	perception	Perception
408	Hamusit to beg. And here the coin is not much available	perception	Perception
409	currently. I'm traveling all over the nearby area because	perception	Perception
410	this doesn't cover me. I'll go as far as Marawi. Why am I	perception	Perception
411	going to sit there on Saturday and be given a nice dress?	perception	Perception
412	and then I come to the shop and sell the nice selected	perception	Perception
413	clothes. Then I pay for the rented house. Having of many	perception	Perception
414	clothes, what do to me?	perception	Perception
417	Respondent: Street life is too bitter to me. When the first	perception	Perception
418	rain is coming, summer is coming, I always think about;	perception	Perception
419	What do I have to pay for rent house? Instead of thinking	perception	Perception
420	of food. My I think all night without sleeping. First of	perception	Perception
421	all, it's a human house. If I did not pay, they take my	perception	Perception

LineNo	Text	Codes	Synthesis 1
422	clothes off, even they don't go out me simply.	perception	Perception
435	And we're not happy about that. By begging, children are	perception	Perception
436	better off. But they do not give it to us. Why? When we beg,	perception	Perception
456	Respondent: Street life is hard for me. It can be nice for	perception	Perception
457	boys. Especially, it is very difficult for a girl. When we	perception	Perception
461	we will raise your child". Even, when we ask them to join	lack of support, perception	Perception
462	in people's house as servant itself. And a girl's life is	lack of support, perception	Perception
463	hard on the road. When we ask, they said, why not you do	lack of support, perception	Perception
477	YW03: The worst part is that, first, I don't have sleep at	perception	Perception
478	night. Men come. And we sit down at night and sleep during	perception	Perception
479	the day, and then we beg. Even, they are saying, "why not	blaming, perception	Perception, Psych
480	do to eat ?" And street life is very difficult. First, my	blaming, perception	Perception, Psych
491	Respondent: Motherhood is very difficult. Um, I think the	perception	Perception
492	males do not care about their children. Even if my husband	perception	Perception
493	is not here, of course, I observe others father here around.	perception	Perception
494	And a girl suffers a lot. Breastfeeding is very painful at	perception	Perception
497	street life is very difficult for a girl, especially for a	perception	Perception
498	mother.	perception	Perception
648	Respondent: on street means is very bad. My child is not	perception	Perception
649	comfortable. The child is hurt very much after we came from	perception	Perception
654	hurt very much. It is not comfortable enough. But, for the	perception, street life	Perception
670	Respondent: coming home with my child without any problem.	perception	Perception
671	What other is there?	perception	Perception
721	in my house. Due to displacement, I hurt in many things.	perception, previous work	Perception
799	will not be paid well. For 200 or 300birr!, I don't know	Financial support, perception	Economical well-b
800	this even but I also don't want my child to be hurt. I want	perception	Perception
801	to pass this day like this, to rare/grows up/ my child. If	hope in God, perception	Perception, Spritul
825	living by saying thanks to God. Like this, sleeping with	hope in God, perception	Perception, Spritul
826	different things rests on you is not the same (??????? ???	perception	Perception
827	??? ????? ??????? ??? ?????).	perception	Perception
834	if God is willing, it becomes bigger. A little work is	Desire to make money, hope in God, perception	Motivation to leave
835	better than sitting in the street throughout the day.	perception	Perception

LineNo	Text	Codes	Synthesis 1
884	Respondent: Ere (??)…I am thinking nothing; I'm just saying	perception	Perception
885	the street life is better. We are new. Street life is that	perception	Perception
886	much, it is somewhat good. It is better than tiredness from	perception	Perception
887	washing clothes and other things.	perception	Perception
902	Respondent: We are new, we don't know anything yet. Street	perception	Perception
903	life is actually difficult but now he is sending money. Now	perception	Perception
904	I sleep on the street since this my arm has broken.	illness, perception	Perception, Physic
976	Respondent: Ere (??)…, I'm fine. Just I am here [on the	no health problem, perception	Perception, Physic
977	street] for short periods until we are familiar with the	perception	Perception
978	area. We think that it is good from nothing. We will be on	perception	Perception
1081	Respondent- Because of the sun or rain, I don't spend the	perception	Perception
1082	whole day on the street. I can get 50birr or 60birr per day	perception	Perception
1084	varies from day to day because I don't spend full time on	Financial support, perception	Economical well-b
1085	the street with the reason of my children i.e. I don't	Financial support, perception	Economical well-b
1086	spend on the street from morning to night like others	perception	Perception
1087	because there is sun or rain.	perception	Perception
1090	Respondent- I usually stay 6 to 7 hours.	perception	Perception
1097	Respondent- Street life is bad, but what's good? It's	perception	Perception
1098	horrible to see a human face, one swears and the other	perception	Perception
1099	insults me that “where will she go holding these children?”	perception	Perception
1100	what is her life direction? Some persons say “she cannot	perception	Perception
1101	work with these children in the light of her life”. In	perception	Perception
1102	general, for me, it's just a bad task. I think… It's	perception	Perception
1103	horrible.	perception	Perception
1105	Respondent- I don't have… but for me, it's just bad in	perception	Perception
1106	general.	perception	Perception
1144	Respondent: paying house rent…, if his own safety is fine	fear of accident	Emotional well-bei
1145	(?? ??? ???… ?? ??? ???? ??? ???). My usual concern is that	fear of accident	Emotional well-bei
1146	one day he may get into a car and sustain accidents. This	fear of accident	Emotional well-bei
1147	kind of disease doesn’t say today and tomorrow (doesn’t	fear of accident	Emotional well-bei
1148	give time). First, if he may find water and will drown	fear of accident	Emotional well-bei
1149	into it or he may get fire and will fall into the fire.	fear of accident	Emotional well-bei
1327	for me (???? ???? ?????? ??? ?? ?? ?? ??????). Unwise man	perception, Praying	Perception, Spritul
1328	thinks that it by my interest. But there is nothing such a	perception	Perception
1329	disgusting thing. “In the name of the Father, and of the	perception, Praying	Perception, Spritul

LineNo	Text	Codes	Synthesis 1
1397	Answer: There is no solution. But it is used for the day to	food shortage, perception	Basic need related
1398	day meal.	food shortage, perception	Basic need related
1400	Answer: Everything is hard, we eat like bread and enjara as	food shortage, perception	Basic need related
1401	we accessed at the time	food shortage, perception	Basic need related
1403	Answer: Everything is hard. For instance, the floor of the	perception	Perception
1404	dormitory is 20 birr a day. But when we were worried, we	perception	Perception
1405	went to church to survive.	perception	Perception
1417	Answer: I did not sleep at night in the street. I dislike	perception, sleeping place	Basic need related
1418	living with beggar.	perception	Perception
1420	Answer: I haven’t encountered a problem. There is no child	perception	Perception
1421	care. There is no care in the dust. There is no washing. It	hope in God, perception	Perception, Spritul
1477	no friends. I have nothing. I just fear for my life. The	family loss, fear for life	Emotional well-bei
1506	Answer: yes, there is, some people says the beggar. But I	Insult, patience, perception	Perception, Psych
1507	can handle it by being patience. There are so many types of	Insult, patience, perception	Perception, Psych
1508	insults. Street life needs being patience. It’s hard to say.	Insult, patience, perception	Perception, Psych
1521	husband], I will leave the street life. The street work	desire to leave, perception	Motivation to leave
1522	itself is very disgusting. It's a shame. It is an insult	perception	Perception
1523	for the family.	perception	Perception
1527	Answer: What can I take with me? What can I eat? What	hopelessness, perception	Emotional well-bei
1528	shall I go out to eat? But when I am in trouble, I kept	hopelessness, perception	Emotional well-bei
1529	silent; and continue this street life.	hopelessness, perception	Emotional well-bei
1547	Answer: Street work is disgusting.	perception	Perception
1575	Answer: When work is lost, I find a way out; there are	perception	Perception
1576	insults on the way. I am not happy. I can't raise my child,	perception	Perception
1577	so I and my child have to fast sometimes. My money is not	perception	Perception
1578	enough. The rent is 600 Birr/month. Now, we wear clothes	perception	Perception
1579	that we bought before street life. Some individuals give	perception	Perception
1580	clothes to my child. The place is uncomfortable.	perception	Perception
1586	Answer: I'm afraid of my child being stolen since she	child protection, poorness	Basic need related
1587	approaches with others by calling my father and my mother.	child protection, poorness	Basic need related
1588	I'm afraid she'll be crashed by a car. There is no money	child protection, modern treatment, poorness	Basic need related
1596	Answer: I am fine. It is my poorness that has affected me.	perception	Perception

LineNo	Text	Codes	Synthesis 1
1664	Respondent: uh…how this could be a life at all, during day	perception	Perception
1665	time sun, we are sleeping under plastic tent “Kenda”. We	perception	Perception
1666	are living because a man cannot be buried alive. I am	perception	Perception
1667	suffering and my children are also suffering, nothing is	perception	Perception
1668	attractive as you live here.	perception	Perception
1694	uniform exercise books, then he suffered living one time	child education, child sufferring	Basic need related
1695	with one relative and the other time to another relative.	child sufferring	Basic need related
1703	soon, that is it. My children life is also getting	child education, perceived children's life	Basic need related
1704	complicated.	perceived children's life	Perception
1716	Respondent: It is very difficult…	perception	Perception
1718	Respondent: Oh, it is very difficult my dear! It is very	Feels sad, perception	Emotional well-bei
1719	difficult very difficult (Sadness on their faces)	Feels sad, perception	Emotional well-bei
1721	Respondent: As you are here, the madman comes, the wind	perception, tearing	Emotional well-bei
1722	comes, and beast comes, when I takeoff this (pointing	perception, tearing	Emotional well-bei
1723	towards the plastic) the rain gets the children. So, it is	perception, tearing	Emotional well-bei
1724	very difficult (tear is coming in her eyes)…(shedding tears	perception, tearing	Emotional well-bei
1725	on her cheeks)…	perception, tearing	Emotional well-bei
1738	Respondent: They feel cold when the plastic touches them.	child sufferring	Basic need related
1739	When it gets raining, they were about to interrupt eating	child sufferring	Basic need related
1740	their dinner and get sleep. Those childless says child,	child sufferring, Human's endless inquiry	Basic need related
1780	in an open field. Ehh, ehh…when it gets rain, they wear a	child sufferring, family/stepmother's push, Feels sad	Basic need related
1781	white plastic item “Festal” see that white plastic “Festal”	child sufferring	Basic need related
1782	placed at the side of the bed (The respondent pointing with	child sufferring	Basic need related
1783	her finger towards the plastic item “Festal”) and we used	child sufferring	Basic need related
1784	to wear when we sleep. And when it happens to this [her	child sufferring	Basic need related
1785	small daughter] I feel sad.	child sufferring	Basic need related
1838	they sleep with me tearing and wearing their “Festal”. They	child independence	Perception
1839	are young and they can go out and live like everyone else	child independence	Perception
1840	but they didn’t do that not to disrupt my life.	child independence	Perception
1846	Respondent: They always question saying why we are here.	inquire why on street	Perception
1847	Why we are here, how? if we have no a judge to hear us, we	inquire why on street	Perception
1848	better go to the countryside to live there.	inquire why on street	Perception
1889	happening to us. On top of that, this [girl] is growing. My	child sufferring, perceived children's life	Basic need related
1890	life is ruined and my children’s life is ruined, otherwise.	child sufferring, perceived children's life	Basic need related

LineNo	Text	Codes	Synthesis 1
1893	Respondent: now she is under our protection, where could	family protection, perceived children's life	Basic need related
1894	they go jumping over us?	family protection, perceived children's life	Basic need related
1902	and want to rape, but this times it is not AIDS that the	fear of geting disease, protection from children, rape intention	Emotional well-bei
1903	only disease, there are many patients with the other	fear of geting disease, rape intention	Emotional well-bei
1904	disease. For example, now I feeling warm since you are here	fear of geting disease, perceived risks	Emotional well-bei
1905	with me because if some problem happen you can protect me.	perceived risks	Perception
1906	But if you are not here who can face the problem? He goes	perceived risks	Perception
1907	killing me or do like that or he just uses me and get rid	perceived risks	Perception
1908	of loading his problems on me. This is what I have been	perceived risks	Perception
1909	through for the past two months. This is not life. I am	perceived risks	Perception
1910	living here leaving a suffering for the public and the	perceived risks	Perception
1911	Christians, my suffering is beyond the public capacity to	perceived risks	Perception
1912	hold me. They cried/teared for me and say to me don’t worry	neighbour's support, perceived risks	Perception
1930	understood/picturized like this. There is no time the poor	perception about officials, poors not heard	Perception
1931	is heard; either I have no one by my side or I have no	poors not heard	Perception
1932	money, I am unheard and facing the problem on my own.	poors not heard	Perception
1944	will not offer you a house for the second time. Off course,	poors not heard, registry as poorest	Perception
1945	they were right at that time, but now they should	poors not heard	Perception
1946	understand me. Now, why not they see my problem in other	poors not heard	Perception
1947	way round and understand me? I am human. If I am not a	poors not heard	Perception
1948	citizen now, I will be sent by a car to my own country. The	Feeling, perception, poors not heard	Emotional well-bei
1949	days are against and the hours (everything) are against me,	Feeling, perception	Emotional well-bei
1950	otherwise no one deserves this live leave alone a person	Feeling, perception	Emotional well-bei
1951	like me who have children; they are the one who takeover	Feeling, investing on children, perception	Emotional well-bei
1957	honors a country. So I have no hearing. They should see my	Everybody's contribution, investing on children, poors not heard	Motivation to leave
1960	benefited by tomorrow. But no one understand me. Though the	investing on children, poors not heard	Motivation to leave
1961	villagers understand me, this [kebele and judge] would not	poors not heard	Perception
1962	understand me.	poors not heard	Perception
1980	way-outs and I sat down asking him for way-outs. Even, you	poorness, poors not heard	Economical well-b
1981	[Interviewers], persons with grace “Nebsi Yalachihu”, are	poors not heard	Perception
1982	the first who entered and talked to us. No one says to me	poors not heard	Perception
1983	it is just like this while the villagers are worrying about	poors not heard	Perception
1984	me. And I can’t do anything. I wish God to bring a	hope in God, poorness, poors not heard	Economical well-b
2037	even with empty stomach. A house to live is my worry. This	perception, support needed to leave	Motivation to leave
2038	[the Kenda tent] is a chicken coop but it is not a human	perception	Perception
2039	shelter. We get this even because of these blessed	perception, will of neighbours	Perception, Social

LineNo	Text	Codes	Synthesis 1
2047	fitch and drink from Abay. My biggest worry is house	child sufferring, suggest change living place	Basic need related
2048	because when the rain water is flowing here [pointing to	child sufferring	Basic need related
2049	the plastic] as they put in as a skirt and the water fall	child sufferring	Basic need related
2050	on here [in the bed] and you can’t imagine the tear they	child sufferring	Basic need related
2051	are tearing and my children’s conscience. On top of that,	child sufferring	Basic need related
2052	they are young and it is very hard. And the son [older	child sufferring	Basic need related
2053	son] is no longer living with me and he can't afford to buy	child sufferring	Basic need related
2054	a house for me either.	child sufferring	Basic need related
2056	Respondent: The son, yes. Besides, they just eat but the	child sufferring	Basic need related
2057	children's conscience is only affected by the house. If you	child sufferring, problem of homelessness	Basic need related
2099	Respondent: What can I describe? It is what I get with	Income source, perception	Economical well-b
2100	suffering and begging daily. From what I get from people;	Income source, perception	Economical well-b
2101	half is for child, and half is also for family. Nothing I	Income source, perception	Economical well-b
2102	can do more than this.	Income source, perception	Economical well-b
2129	Respondent: So true! I used to live a better life there	perception	Perception
2130	[Jimma] than the life here. But I left saddened there	fear of children loss, perception	Emotional well-bei
2131	[Jimma] and I came up with the idea that these people would	fear of children loss, perception	Emotional well-bei
2132	destroy my children. Otherwise, that one was better for me.	fear of children loss, perception	Emotional well-bei
2133	But I came begging not to lost my children’s life since I	fear of children loss, husband loss	Emotional well-bei
2134	lost their father.	fear of children loss, husband loss	Emotional well-bei

Synthesis 1: Motivation to leave

LineNo	Text	Codes	Synthesis 1
	Document: IDI-Translation_Compiled		
71	difficult for me to move and walk easily. I believe that	desire for education, mobility difficulty	Motivation to leave,
72	this child (she is pointing to the older child) should join	desire for education	Motivation to leave
73	school, but I do not know the place. When he come back from	desire for education	Motivation to leave
84	hold him there, and he will learn and collected,	education for reinforcement	Motivation to leave
85	“Yeshekefal”, there.	education for reinforcement	Motivation to leave
215	Respondent: If I get, what am I going to do in the street?	desire to leave	Motivation to leave
216	Stomach does not let us one day even we eat in the morning.	desire to leave	Motivation to leave
217	If I get a small dormitory, food for feeding children, I	desire to leave	Motivation to leave
218	could put “Kesha” in it. It would be great if I could feed	desire to leave	Motivation to leave
219	the children and feed them off the street. I will feed them	desire to leave	Motivation to leave
220	at home by balancing the food, they may eat less than they	desire to leave	Motivation to leave
221	can eat at street. I spread the children on the streets	desire to leave	Motivation to leave
225	suspect it crushes them. I don't want to sit on the street	desire to leave, fear of accident	Emotional well-bein
226	if I find five or six zinc cans and a snack for them.	desire to leave	Motivation to leave
228	Respondent: Ehh…I have nothing. My idea now is, if I don't	desire to give child	Motivation to leave
229	recover, with this age, by the age of 30, I beg, I plan to	desire to give child	Motivation to leave
230	take over this big child to rural village and I turn around	desire to give child	Motivation to leave
231	and fall of a church, a monastery with a little child.	Joining monastries	Motivation to leave,
232	Where will I go forever in handicap? If there is a better	Joining monastries	Motivation to leave,
252	Respondent: For example, if I find a container. I can’t go	Desire to make money	Motivation to leave
253	outside. So, I sell some items from the container. I sold	Desire to make money	Motivation to leave
254	those items and raised children. Then I can sell it and	Desire to make money	Motivation to leave
255	feed the children. I don't have to bake bread or do day	Desire to make money	Motivation to leave
256	work. I go to the store and sell something. I can work with	Desire to make money	Motivation to leave
257	a clear mind. I have three children, so I can feed and	Desire to make money	Motivation to leave
259	can live with modest children. If they ate one injera	desire for education, Desire to save money	Motivation to leave
260	previously, I will feed half injera and I can teach and	desire for education	Motivation to leave
261	grow them.	desire for education	Motivation to leave
304	Respondent: I just don't want to be on the street. I want	desire to leave, perception	Motivation to leave,
305	to go out looking for a daily labor	desire to leave, perception	Motivation to leave,
311	but I in my own, I was so overwhelmed. Why because sitting	desire to leave, perception	Motivation to leave,
312	here and selling corn is an alternative work. I don't want	desire to leave, perception	Motivation to leave,
313	to beg, but it is a matter of losing to do this. I prefer	desire to leave, perception	Motivation to leave,

LineNo	Text	Codes	Synthesis 1
314	this (selling corn), I don't want the street.	desire to leave, perception	Motivation to leave,
375	I move to other house as servant, no one will hire me	Desire to make money, lack of support	Motivation to leave
425	Respondent: I have no idea what I am going to do now, but I	desire to leave	Motivation to leave
426	accept anything that will get out of us from begging. I	desire to leave	Motivation to leave
427	want to leave, but no choice. It would be nice if I could	desire to leave	Motivation to leave
428	bring some sorghum, and sit here. It is because I am angry	desire to leave	Motivation to leave
429	with begging. If anything from anyone or government give to	desire to leave	Motivation to leave
430	me, I will leave the street.	desire to leave	Motivation to leave
511	maximum effort, “tewutertirie”, and if someone can help me	desire for education, support	Motivation to leave
512	a little. And if I get schools that admit at the morning	desire for education	Motivation to leave
513	and released him at 12 o'clock in the afternoon, I want to	desire for education	Motivation to leave
514	work.	desire for education	Motivation to leave
586	Respondent: Let's get some help. Because we can't change	desire to leave	Motivation to leave
587	working in here. There is no work on this street. And if	desire to leave	Motivation to leave
588	an organization or the government gives us something little,	desire to leave	Motivation to leave
589	we can do it.	desire to leave	Motivation to leave
592	Respondent: I wanted to leave before. And I failed. I	desire to leave	Motivation to leave
593	thought I could start shoeshine. And someone says I'll	desire to leave	Motivation to leave
594	start for you, but he doesn't. I think we will leave if we	desire to leave	Motivation to leave
595	do something else. Someone started shoeshine to me and then	desire to leave, Effort to end street	Efforts to leave, Mo
598	Respondent: At least, if someone starts a small shop, I	desire to leave	Motivation to leave
599	believe I will leave.	desire to leave	Motivation to leave
602	Respondent: Now, for example, if someone rents a house, I	desire to leave	Motivation to leave
603	will leave. If they start a small business to me and give	desire to leave	Motivation to leave
604	me a place for my child to stay, I will leave.	desire to leave	Motivation to leave
606	Respondent: For example, if I get child daily care, the	desire for education	Motivation to leave
607	baby will change. Because children smoke on this street.	desire for education	Motivation to leave
615	Respondent: If the government does something to me, I will	desire for education	Motivation to leave
616	leave. Because the people see us as dirty materials. They	desire for education	Motivation to leave
619	think if the government does something for us, we will	desire to leave	Motivation to leave
620	leave.	desire to leave	Motivation to leave
794	her child, she is living like this”. If I want to work, I	desire to work, Insult	Motivation to leave,
795	can work now. But no one can let me in to his house with my	desire to work, stigma and discrimination	Motivation to leave,

LineNo	Text	Codes	Synthesis 1
819	option. First, now, I for example, if my child grows up and	desire to leave	Motivation to leave
820	becomes like other person’s wise child, I want to engage a	desire to leave	Motivation to leave
821	day work (??? ??) something like that and want to live. I	blaming, desire to leave	Motivation to leave,
832	will teach my child, and I want to be self-sufficient.	desire for education, desire to leave	Motivation to leave
833	Rather than being here over day, if I have a little work,	Desire to make money	Motivation to leave
834	if God is willing, it becomes bigger. A little work is	Desire to make money, hope in God, perception	Motivation to leave,
838	Respondent: I can decide if I have my own little thing, for	Desire to make money	Motivation to leave
839	example, I may start with something like selling potato or	Desire to make money	Motivation to leave
840	onion. But I can't decide by myself unless I have my own	Desire to make money	Motivation to leave
841	thing.	Desire to make money	Motivation to leave
851	Respondent: if I get support, yes, I need to give up the	desire to leave	Motivation to leave
852	street life. One from many, one of the ten individuals	blaming, desire to leave	Motivation to leave,
853	walking on the roadside is blame you, one of the other ten	blaming, desire to leave	Motivation to leave,
854	individuals drop cents to you. Like somebody who drops	desire to leave, support	Motivation to leave
855	cents, who knows, one day on behalf of me or my child, a	desire to leave, support	Motivation to leave
856	person may be told me to do something, if it is so I can	desire to leave	Motivation to leave
857	immediately give up my street life and work, without which	desire to leave	Motivation to leave
858	there is no one to help me.	desire to leave	Motivation to leave
907	due to my hand is broken. I want to work to eat. I want to	desire to work, push factor	Motivation to leave,
908	work in someone's house as a part-timer. For the future, Up	desire to leave, desire to work	Motivation to leave
909	to that, we are here to stay.	desire to leave	Motivation to leave
933	only child living with me. The three children who live with	giving child to relative	Efforts to leave, Mo
934	my mother are 6, 8, and 11 years old. This child is 4 years	giving child to relative	Efforts to leave, Mo
980	periods on the street however, until we are familiar with	desire to leave	Motivation to leave
981	the locality. I don’t like to work on the street for long	desire to leave	Motivation to leave
982	periods.	desire to leave	Motivation to leave
1012	Respondent: until the end of rainy season I am here, and	desire to leave	Motivation to leave
1013	then I will quit it. I will leave it if I find a job.	desire to leave	Motivation to leave
1016	money. We have never tried to leave the street but we hope	desire to leave, push factor	Motivation to leave,
1017	to be progressed, they will give house, we think the	desire to leave	Motivation to leave
1018	government.	desire to leave	Motivation to leave
1270	Respondent: Just only to give up… I'll leave it (???? ??…	desire to leave	Motivation to leave
1271	??? ??????). She [the small child] will be two years old in	desire to leave	Motivation to leave
1276	the small kid regarding fire. She will feed the small kid	clothes support, desire to leave, support from child	Economical well-be
1277	what I give her. Then after, if I get some money, I can	desire to leave	Motivation to leave

LineNo	Text	Codes	Synthesis 1
1278	fried the corn on the cob and sell, I can sell sugarcane	desire to leave	Motivation to leave
1279	and I plan to sell mobile cards even if it doesn't make a	desire to leave	Motivation to leave
1280	profit.	desire to leave	Motivation to leave
1282	Respondent: it is the life… firstly the life, secondly	desire to leave	Motivation to leave
1283	forever here? (??? ??… ???? ???? ???? ?? ??? ??? ????). I	desire to leave	Motivation to leave
1284	and my children are hurt. For me, it is due to my problem	desire to leave	Motivation to leave
1285	but I also hurt my children (??? ???? ?? ???? ??? ?????).	desire to leave	Motivation to leave
1288	Respondent: If I don't show the work for them, they will	desire to leave	Motivation to leave
1289	adopt this task (begging). For example, if I don't enroll	desire for education, desire to leave	Motivation to leave
1290	the elder child in school next year, she will come up with	desire for education, desire to leave	Motivation to leave
1291	my idea (now she is 4 years old).	desire for education, desire to leave	Motivation to leave
1310	Respondent: If they grow up, and if I get money, container,	desire to leave, Desire to make money	Motivation to leave
1311	I can write, I can speak and I can make different things.	Desire to make money	Motivation to leave
1312	If my children are a beat grows up, I can make different	Desire to make money	Motivation to leave
1313	things. I do not live like everyone else (I do not always	desire to leave	Motivation to leave
1314	live like this). If I find anything, they (kids) will know	desire to leave	Motivation to leave
1315	what is right and what is wrong, I can read and write, I	desire to leave	Motivation to leave
1316	can do something.	desire to leave	Motivation to leave
1520	Answer: If I got money, why I am here? If I find him [her	desire to leave	Motivation to leave
1521	husband], I will leave the street life. The street work	desire to leave, perception	Motivation to leave,
1532	Answer: For my son, if he grows up, he will go to school	desire for education	Motivation to leave
1534	Answer: He will Learn and work by his choice.	child work, desire for education	Efforts to leave, Mo
1541	Answer: Something to get started any work. Beggaring is	support needed to leave	Motivation to leave
1542	temporary, not a permanent solution. I need Work. I would	support needed to leave	Motivation to leave
1543	love to work in private. Even, I want to work in a private	support needed to leave	Motivation to leave
1544	company.	support needed to leave	Motivation to leave
1639	Answer: The one who helps my child without separating with	support needed to leave	Motivation to leave
1640	me. For me, I work. I need a job. But for the time being, I	support needed to leave	Motivation to leave
1641	only want to help my child(She had the worst face).	support needed to leave	Motivation to leave
1713	Respondent: Yes	desire to work	Motivation to leave
1741	those who have child says a means to raise a child. I would	desire to own house, Human's endless inquiry	Motivation to leave
1742	not ask for what to eat if I had an entrance to my neck	desire to own house	Motivation to leave
1743	[This is to mean that a house to live in].	desire to own house	Motivation to leave
1850	Respondent: They are insisting me to let get out of here	suggest change living place	Motivation to leave

LineNo	Text	Codes	Synthesis 1
1851	and go to a rural town if I can't afford living here	suggest change living place	Motivation to leave
1852	otherwise. They just sit here and do their daily homework	children's worry, suggest change living place	Emotional well-bein
1951	like me who have children; they are the one who takeover	Feeling, investing on children, perception	Emotional well-bein
1952	their country, they are useful and the one who can	investing on children	Motivation to leave
1953	contribute much by tomorrow though I am not us such	investing on children	Motivation to leave
1954	important. Even I may be also useful by enclosing myself	investing on children	Motivation to leave
1957	honors a country. So I have no hearing. They should see my	Everybody's contribution, investing on children, poors not heard	Motivation to leave,
1958	children, they have so many things, it mirrors their	investing on children	Motivation to leave
1959	tomorrow [they have bright future], and they will be	investing on children	Motivation to leave
1960	benefited by tomorrow. But no one understand me. Though the	investing on children, poors not heard	Motivation to leave,
1965	Respondent: I just want to see him self-contained and get	desire for self-contained life	Motivation to leave
1966	relieved for worrying with me and live relaxed life. If	desire for self-contained life	Motivation to leave
1967	they live such a life, I would say thank you my lord you	desire for self-contained life	Motivation to leave
1968	are not let go. I did not say this as a challenge, I did	desire for self-contained life	Motivation to leave
1969	not take it. And I didn't say this [street life] a	desire for self-contained life	Motivation to leave
1970	challenge, I will not say it again, I just keep saying	desire for self-contained life	Motivation to leave
1973	them work there. Today, ehhh, if he trades chickens, ehhh,	desire to work, hopelessness	Emotional well-bein
1974	if he sells fattened cattle, he can be a trader. But leave	desire to work, poorness	Economical well-be
1975	alone affording that I am homeless and I let them sleep on	desire to work, poorness	Economical well-be
1987	feed themselves, what can I say to other this? Now this	desire to work, hope in God	Motivation to leave,
1988	[middle boy] one is grade seven, he wants to bring and	desire to work	Motivation to leave
1989	drive BaJaJ [three-legged vehicle] while attending school.	desire to work	Motivation to leave
1990	He wants to do many things. The other one [older boy], he	desire to work	Motivation to leave
1991	wants to fatten cattle. But these all desire are nothing if	desire to work	Motivation to leave
1992	there is no capacity to afford them. A human is, now, for	desire to work	Motivation to leave
1993	example me myself, if I own a house, I make Injera as I	desire to work	Motivation to leave
1994	capable of it, take my tablets and take rest rather than	desire to work	Motivation to leave
1995	waking up at night. Then, I can prepare a place to sit and	desire to work	Motivation to leave
1996	sell near to house and take care of the children. But I	desire to work, poorness	Economical well-be
2002	Respondent: This one [her daughter], when she sees me	children's desire, children's emotional support	Motivation to leave,
2003	tearing like now, she says to me "just leave it my mother I	children's desire, children's emotional support	Motivation to leave,
2004	will go to America and buy for you a house, don't worry."	children's desire, children's emotional support	Motivation to leave,
2005	That one [her son] also says "I will carry a sac and you	children's desire, children's emotional support	Motivation to leave,
2006	will rent a small house "slum house" at far place and live.	children's desire	Motivation to leave
2017	Respondent: Just to get out of this life…just for my neck	support needed to leave	Motivation to leave
2018	to let enter (this is to mean a house to live), just about	support needed to leave	Motivation to leave
2019	the size of a kitchen, a wealthy man's kitchen house sized,	support needed to leave	Motivation to leave
2020	that is all. But for the rest, I can live by working or	support needed to leave	Motivation to leave

LineNo	Text	Codes	Synthesis 1
2021	begging. The most urgent and priority issues and which	support needed to leave	Motivation to leave
2022	became a conscience wound for my children is a house to	support needed to leave	Motivation to leave
2023	live “Anget Masgebia”. Otherwise, since their father death	lived work experiences, support needed to leave	Motivation to leave
2026	brewing and baking to raise my children. But now, the	lived work experiences, support needed to leave	Motivation to leave
2027	urgent issue is house to live; if I want to have rental	stigma and discrimination, support needed to leave	Motivation to leave,
2034	if I get a house to live, where there is no one used to	support needed to leave	Motivation to leave
2035	live in, where there is nothing to round it, I embrace/hold	support needed to leave	Motivation to leave
2036	my children and raise and educate them as much as I can	support needed to leave	Motivation to leave
2037	even with empty stomach. A house to live is my worry. This	perception, support needed to leave	Motivation to leave,
2045	Respondent: There is nothing worse than a house to live. I,	suggest change living place	Motivation to leave
2046	even, can go in a place with no access to water, I can	suggest change living place	Motivation to leave
2047	fitch and drink from Abay. My biggest worry is house	child sufferring, suggest change living place	Basic need related,
2176	Respondent: I will send her to school in the future.	desire for education	Motivation to leave
2178	Respondent: I will send her to school in the future even by	desire for education	Motivation to leave
2179	begging. I will even send this [little child] himself if he	desire for education	Motivation to leave
2180	grows for me. But now, he unable to maintain upright	child disability, desire for education	Motivation to leave,
2181	position and walk/go. Thank God for another problem.	child disability, desire for education, Thanking God	Motivation to leave,
2278	Respondent: I want to give up street life if I get job and	desire to leave	Motivation to leave
2279	do a job. (Uh) If I get a job, I'll work if the child is	desire to leave	Motivation to leave
2280	healthy; What will it do for me now to sit here and remain	desire to leave	Motivation to leave
2281	passive? I would rather work and eat.	desire to leave	Motivation to leave
2289	Respondent: I want her to get an education and get a job.	desire for education	Motivation to leave
2293	Respondent: For this, if he grows up for me, I want him to	desire for education	Motivation to leave
2294	get educated either from formal education or from priest. I	desire for education	Motivation to leave
2295	want him continue and grow up with either of the two	desire for education	Motivation to leave
2296	[formal or informal/priest].	desire for education	Motivation to leave
2299	Respondent: I need help, I want a house. (Eh) If I get, I	support needed to leave	Motivation to leave
2300	want a house	support needed to leave	Motivation to leave
2302	Respondent: Ehh, I also want some items and also clothes	support needed to leave	Motivation to leave
2303	since I don't have any clothes.	support needed to leave	Motivation to leave
2306	Respondent: If I am given, I want some amount of money for	support needed to leave	Motivation to leave
2307	the child's upbringing and I want a house.	support needed to leave	Motivation to leave
2312	Respondent: Aha! It is what are we talking about since then;	support needed to leave	Motivation to leave

LineNo	Text	Codes	Synthesis 1
2313	clothes, utensils/material such kinds of support. I accept	support needed to leave	Motivation to leave
2314	what people given me thinking for their soul. Other than	support needed to leave	Motivation to leave
2315	this, what more could I ask for? This is it.	support needed to leave	Motivation to leave

Synthesis 1: Efforts to leave

LineNo	Text	Codes	Synthesis 1
	Document: IDI-Translation_Compiled		
76	Respondent: He did not start school. He goes to Priest’s	child education	Basic need related, Efforts to leave
77	school some times. But for “migtosh” purpose he goes to	child behavior, child education	Basic need related, Efforts to leave
185	children walking around. The that child [oldest child] is	Effort to end street, Need adult protection	Efforts to leave, Perception
186	more mature than them, I gave her to someone else. Michael	Effort to end street, Praying	Efforts to leave, Spritul well-being
330	She choked my throat. She lives of the street. She was	child education, Chocked throat	Basic need related, Efforts to leave, Physi
331	grade seventh here in Sertse. She said that “learning would	child education	Basic need related, Efforts to leave
332	not help me.” I told the police, “He said that if she is	child education	Basic need related, Efforts to leave
333	above you, you can't do nothing for her." Instead of this,	child education	Basic need related, Efforts to leave
360	Respondent: This one (she is pointing to the middle child)	child education	Basic need related, Efforts to leave
361	is in. She (she is pointing to little child) is still young.	child education	Basic need related, Efforts to leave
362	I tried much too, but they refuse me to in her due to her	child education	Basic need related, Efforts to leave
363	childness.	child education	Basic need related, Efforts to leave
508	Respondent: If I have a job in the future, I would like to	child education	Basic need related, Efforts to leave
509	work; rented dormitory and I want to teach my child in	child education	Basic need related, Efforts to leave
510	kindergarten. If someone helps me a little. I will use my	child education, support	Basic need related, Efforts to leave
595	do something else. Someone started shoeshine to me and then	desire to leave, Effort to end street	Efforts to leave, Motivation to leave
596	I stopped this because no income from it.	Effort to end street	Efforts to leave
933	only child living with me. The three children who live with	giving child to relative	Efforts to leave, Motivation to leave
934	my mother are 6, 8, and 11 years old. This child is 4 years	giving child to relative	Efforts to leave, Motivation to leave
1028	Respondent: Ere… I didn’t think ever, thanks to God. Up to	Effort to end street, Thanking God	Efforts to leave, Spritul well-being
1029	now he is fine; he is managing us, I didn’t think ever.	Effort to end street	Efforts to leave
1534	Answer: He will Learn and work by his choice.	child work, desire for education	Efforts to leave, Motivation to leave
1535	Interviewer: Have you ever tried any thing for him?	child work, hope in child	Efforts to leave
1536	Answer: he will help me to leave this (street) life and	child work, hope in child	Efforts to leave
1537	after he matured he will work his work.	child work, hope in child	Efforts to leave
1684	else and the children are also students.	child education, low income for livelihood	Basic need related, Economical well-bein
1686	Respondent: One is not attending school	child education	Basic need related, Efforts to leave
1688	Respondent: Yes, those you got them in the morning are	child education	Basic need related, Efforts to leave
1689	in-school	child education	Basic need related, Efforts to leave

LineNo	Text	Codes	Synthesis 1
1693	Respondent: unable to send that one to school I can’t able	child education	Basic need related, Efforts to leave
1694	uniform exercise books, then he suffered living one time	child education, child sufferring	Basic need related, Efforts to leave, Perc
1697	Respondent: Oh, I never let him to start because I can’t	child education	Basic need related, Efforts to leave
1698	afford.	child education	Basic need related, Efforts to leave
1700	Respondent: Yes, but now he is insisting me to send him.	child education	Basic need related, Efforts to leave
1701	When he come, I am insisting him not start by telling him	child education	Basic need related, Efforts to leave
1702	as I am getting tired and our living condition here and	child education	Basic need related, Efforts to leave
1703	soon, that is it. My children life is also getting	child education, perceived children's life	Basic need related, Efforts to leave, Perc
1706	Respondent: What do they do?	child work, no job	Efforts to leave, Push factors
1708	Respondent: What job is there? This time, there is no job	child work, no job	Efforts to leave, Push factors
1709	to involve, you can’t get a job. You may have interest but	child work, no job	Efforts to leave, Push factors
1710	there is job or you can’t find a job.	child work, no job	Efforts to leave, Push factors
2165	Respondent: not in school; I brought her dropping her	child education	Basic need related, Efforts to leave
2166	education.	child education	Basic need related, Efforts to leave
2168	Respondent: Yes	child education	Basic need related, Efforts to leave
2170	Respondent: she was grade 1. She was learning in Affaan	child education	Basic need related, Efforts to leave
2171	Oromo and she did not progress well.	child education	Basic need related, Efforts to leave
2173	Respondent: Now, she is not in school since we are here	child education	Basic need related, Efforts to leave
2174	because the time has passed.	child education	Basic need related, Efforts to leave
2283	Respondent: Me… What am I trying to do? To work and eat,	Effort to end street	Efforts to leave
2284	the boy can’t stand up and walk for me, how can I do that?!	Effort to end street	Efforts to leave
