# Supplementary material for: Street mothers’ well-being and motivation to leave street life in Bahir Dar city, Ethiopia: A phenomenological study
Source: PLoS One. 2022 Dec 15;17(12):e0278612. doi: 10.1371/journal.pone.0278612 (PMC9754257; doi:10.1371/journal.pone.0278612)
Supplement: Attachment — Submitted filename: REVIEWER REPORT-19Sept2022.docx [file pone.0278612.s004.docx]

*PLOS ONE*

***MANUSCRIPT NUMBER:*** *PONE-D-22-20787*

**TITLE:** *Street Mothers’ Well-Being and Motivation to Leave Street Life in Bahir Dar City,*

*Ethiopia: A Phenomenological Study*

REVIEWER REPORT

1. **Summary of paper**

This paper explores the well-being of street mothers and their children in Bahir Dar City, Ethiopia. It addresses an important topic, especially given that the perspectives of street mothers is significant input for policy makers and implementers working to improve the well-being of the vulnerable in Ethiopia. Using the phenomenological approach as a method, the authors found out that street mothers have poor wellbeing status in almost all different domains of wellbeing the study sought to explore. The study outlined life challenges such as

lack of safe sleeping space at night, increased vulnerability of their children to road traffic, lack of access to basic needs were all contributing factors of poor wellbeing among street m others. The authors added that despite the challenges faced by the street mothers, the desire to educate and invest in their children, desire to live a satisfying were among the motivational pushing them. The authors concluded that the mothers are unable to achieve these goals despite their efforts, hence the need for an assistance mechanism by stakeholders such as Labour and Social Affairs of Amhara region and Regional Health Bureau to support them.

The writing is clear and structure of the paper satisfying.

The main strength of this paper is that it addresses wellbeing of street mothers in holistic way. It gives an insight into the different components of wellbeing and their interconnectedness. As such this article represents a good study which has the potential influence the thinking of policy makers about addressing the wellbeing of the vulnerable holistically. Some of the weaknesses is that the study does not provide a thorough explanation of the various domains of wellbeing explored in the study. The linkages between the challenges faced by the respondents and the various domains of wellbeing.

2. **Main comments**

**Line 27-28**: What is perception of street mothers about street life? The results section of the abstract does not capture that. Shed a light on that.

**Line 65-66**: “This made to have many people lead their livelihood on street overtime.” This sentence does not make sense. Articulate properly the linkage between many Ethiopians living in poverty and streetism.

**Line 79:** Rephrase sentence in line 79. The evidence provided from line 80 to 83 to support the claim that street people are on the rise is not clear. Before concluding that street people are on the rise there should be a comparison of data between previous year and current year. You could perhaps either look at data either before or after 2018 and compare with 2018.

**Line 87-88:** The phrasing is a bit unclear. Should read: *For mothers with children, the lack of secure accommodation or creche facilities cannot allow* ***them*** *to move far from home to look for income opportunities.*

***Line 118-123:*** *provide rationale for choosing Bahir Dar city. What makes the site ideal for your study? Is the phenomenon of streetism prevalent in the city? Any figures to support that?*

**Line 137-139:** Perhaps you could break down the interview guide into themes. It could be in the form of a short table to give readers an insight of the kind of questions asked during the interview. It could also be an appendix.

***Line 131-133:*** Expatiate the process of the sampling selection. How did the authors select respondents. Did authors use snowball? Detail the steps used to select mothers from different age groups. Provide *enough information to validate the study and make it easy for another researcher to reproduce the study with the same methods.*

**Line 180-184:** I suggest the thematic framework developed during analysis be added as an appendix if possible.

**Line 218-229:** These quotes do not necessarily represent the theme of physical wellbeing of street mothers highlighted. For instance, how does the physical problems of their husbands/children impact street mothers. If does impact them, would it not impact their mental wellbeing rather than physical wellbeing?

**Line 245-247:** Is streetism a causal factor of this sentiment by family members or because of another factor

**Line 252-256:** I struggle to understand how the quotes provided is related to a bad relation with family and therefore affecting their social wellbeing.

**Line 311-345:** I will suggest that emotional wellbeing and mental health are combined into one grand theme. The quotes/sub-themes under the two themes are almost the same and will be better to have them under one theme.

**Line 380:** Provide a context of what the holy water is. It would be good to enlighten your readers what ‘attending holy water’ means.

**Line 553-Line 556:** There seems to be a disconnect between line 553 to line 555 and the second part of line 555 to line 559. How are the broader social conditions linked to social well-being of your respondents? Need to make it clear

Line 576-577: Did the study cited from lines 576-577 establish a link between human connection with God and well-being? Perhaps you could discuss the study cited from that angle and link it to your current study on spiritual well-being.

3. ***Minor comments***

***Line 129-130:*** *“The researchers were also made discussion each day after interview.” Check grammar*

**Line 157-158:** “*Data were read and re-read by the research team until understanding the full content of the response.” Check grammar*

**Line 192-195:** Check grammar and tenses

**Line 200: “***The age of street mothers participated in the study rage from 20 to 42 years.”* “rage” should be ranges

**Line 222**: “*Street mothers also stated as their children had physical health problem.”* Check tenses

**Line 380:** “holly water” Check spellings

**Line 514-516: “***Some street mothers have tried different mechanisms to end the street life such as educating their children, giving their children to their relatives and else persons, and let them devote their time on work and education.”* Check spellings and tenses

Line 521-522: “*Another a 40 years old mother described how she have tried her best to educate her child and how she was challenged by her child.”* Check spellings and tenses
